# A spatial transcriptomic signature of 26 genes resolved at single-cell resolution characterizes high-risk gastric cancer precursors

**DOI:** 10.1038/s41698-025-00816-w

**Published:** 2025-02-25

**Authors:** Robert J. Huang, Ignacio A. Wichmann, Andrew Su, Anuja Sathe, Miranda V. Shum, Susan M. Grimes, Rithika Meka, Alison Almeda, Xiangqi Bai, Jeanne Shen, Quan Nguyen, Ingrid Luo, Summer S. Han, Manuel R. Amieva, Joo Ha Hwang, Hanlee P. Ji

**Affiliations:** 1 Division of Gastroenterology, Department of Medicine, Stanford School of Medicine, Stanford, CA 94305 USA; 2 Division of Oncology, Department of Medicine, Stanford School of Medicine, Stanford, CA 94305 USA; 3 Division of Obstetrics and Gynecology, Department of Obstetrics, Escuela de Medicina, Pontificia Universidad Católica de Chile, Santiago, 8331150 Chile; 4 Advanced Center for Chronic Diseases (ACCDiS), Pontificia Universidad Católica de Chile, Santiago, 8331150 Chile; 5 Institute for Molecular Bioscience, The University of Queensland, Brisbane, QLD 4072 Australia; 6 Department of Pathology, Stanford School of Medicine, Stanford, CA 94305 USA; 7 Quantitative Sciences Unit, Department of Medicine, Stanford School of Medicine, Stanford, CA 94305 USA; 8 Department of Neurosurgery, Stanford School of Medicine, Stanford, CA 94305 USA; 9 Stanford Cancer Institute, Stanford, CA 94305 USA; 10 Department of Microbiology and Immunology, Stanford University, Stanford, CA 94305 USA; 11 Department of Pediatrics, Stanford University, Stanford, CA 94305 USA

**Keywords:** Predictive markers, Gastric cancer

## Abstract

Gastric cancer precursors demonstrate highly-variable rates of progression toward neoplasia. Certain high-risk precursors, such as gastric intestinal metaplasia with advanced histologic features, may be at up to 30-fold increased risk for progression compared to lower-risk intestinal metaplasia. The biological differences between high- and low-risk lesions have been incompletely explored. In this study, we use several clinical cohorts to characterize the microenvironment of advanced gastric cancer precursors relative to low-risk lesions using bulk, spatial, and single-cell gene expression assays. We identified a 26-gene panel which is associated with advanced lesions, localizes to metaplastic glands on histopathology, and is expressed in aberrant mature and immature intestinal cells not normally present in the healthy stomach. This gene expression signature suggests an important role of the immature intestinal lineages in promoting carcinogenesis in the metaplastic microenvironment. These findings may help to inform future biomarker development and strategies of gastric cancer prevention.

## Introduction

Gastric cancer (GC) is a leading source of global cancer morbidity and mortality^1^. Survival from GC both worldwide and in Western nations remains poor (under 35%)^2^, due to advanced stages at time of diagnosis. The intestinal subtype makes up a substantial majority of GCs and follows a carcinogenic pathway termed Correa’s cascade^3^. This premalignant evolution involves the gastric mucosa progressing through a series of histopathologic changes: non-atrophic gastritis (NAG), chronic atrophic gastritis (CAG), gastric intestinal metaplasia (GIM), dysplasia and ultimately GC.

GIM provides an opportunity for cancer interception, as it often persists as a distinct lesion with unique characteristics and poses a continued risk for GC. As a result, GIM lesions are easily recognizable on upper endoscopy. The prevalence of GIM is estimated to be 5–10% of the general population in Western nations^4,5^. However, only a very small subset of patients with GIM will progress to GC over long-term follow-up^6,7^. Identifying this subset of “high-risk” GIM has become a high clinical priority and may lead to strategies of early detection and mortality reduction. One promising risk stratification tool is the Operative Link on Gastric Intestinal Metaplasia (OLGIM) staging framework^8^. OLGIM scoring represents a composite endpoint which incorporates two features: metaplastic cellularity at the microscopic level and topographic extent at the anatomical level. The cellularity of metaplasia may be assessed using a visual-analog scale^9^, or by manual annotation of the percentages of glands involved^10^. The cellularity within each of the two major regions of the stomach, the antrum and body, are calculated. The antral and body scores are then used to calculate the overall summary score, which ranges from zero (no GIM) to four (highest risk). Recent studies have demonstrated that advanced OLGIM lesions (Stages III or IV) are 25–34 times more likely to progress to high-grade dysplasia or GC compared to early OLGIM lesions (Stage I)^11,12^. As such, while both high- and low-OLGIM lesions are both precursors to GC, their natural history and clinical management are radically different.

Most prior translational studies of GIM have not differentiated between high- and low-risk status. This is in part because scoring systems such as OLGIM have only recently been developed^8^. Another reason may be the manually-intensive nature required to clinically stage specimens using OLGIM, which requires both a dedicated biopsy protocol and pathologists with skill and experience in interpreting histologic severity^9^. The OLGIM staging system has recently been endorsed by a number of international guidelines^13,14^; as such, their use will increase worldwide in the coming years. There exists a critical need to better understand molecular and cellular differences between lesions on either end of this spectrum.

Using an OLGIM-staged cohort, we identified a gene expression signature which characterizes advanced, high-risk GIM lesions (defined as OLGIM III or IV). This analysis used an integrated multi-omics approach that included conventional RNA sequencing (RNA-seq), spatial transcriptomics analysis, single-cell RNA sequencing (scRNA-seq), and single-molecule fluorescent in situ hybridization (smFISH). In summary, we discovered a spatially-mapped high-risk gene expression signature which characterizes advanced GIM lesions, is shared by intestinal-type GCs, and localizes to aberrant mature and immature intestinal cells within the metaplastic microenvironment.

## Results

### Cohorts

We provide an overview of the specimen sourcing in Supplementary Data 1. The Gastric Precancerous Conditions Study (**GAPS**) is a prospective cohort of individuals undergoing endoscopy who are at increased risk for GC due to presence of symptoms (e.g., dyspepsia, anemia), personal history (e.g., GIM), or family history of GC. Enrolled subjects undergo biopsies according to the updated Sydney System, with standardized histologic assessment allowing for calculation of OLGIM stage and determination of *Helicobacter pyori* (**Hp**) colonization. Sample-level phenotypic data and RNA sequencing metrics can be found in Supplementary Data 2. Notably, while GAPS recruits patients with active and eradicated Hp infection, we chose to develop the bulk expression signature only in patients who were negative for Hp at time of tissue collection in order to remove active Hp gastritis as a confounding variable in the analysis.

The Cancer Genome Atlas Stomach Adenocarcinoma (TCGA-STAD) genomic dataset is comprised of GC samples which had not been previously treated by chemotherapy or radiation prior to genomic analysis^15^. From these samples, we analyzed gene expression data from 180 intestinal-type GC primary tumors and 18 patient-matched tumor-adjacent controls. We obtained the de-identified patient clinical phenotype and RNA-seq results from Genomic Data Commons through TCGABiolinks^16^ R package. Tumor-level phenotypic data (e.g., tumor location) is available in Supplementary Data 3.

We analyzed a scRNA-seq dataset for gastric pathology across Correa’s cascade (normal, NAG, CAG, GIM, and GC). This sample set constituted both de novo scRNA-seq data from prospectively collected samples along with public data sets. In total, the integrated scRNA-seq dataset comprised 40 biopsy samples from 26 patients: two normal controls, three NAG, three CAG, thirteen GIM, nine tumor-adjacent controls, and ten primary gastric tumors. Clinical phenotypic information (specimen location and histology), cell counts, and sequencing metrics are available in Supplementary Data 4.

For the spatial mapping and localization, we used formalin-fixed paraffin-embedded (FFPE) tissues from five patients (four GIM, one GC). The hematoxylin and eosin (H&E)-stained sections were manually annotated by an expert pathologist at the glandular level for regions of normal base, normal pit, metaplasia, dysplasia, and carcinoma. For spatial transcriptomics, unstained sections were placed on the Visium assay slide (10X Genomics) and stained with H&E followed by probe-based sequencing. For the single-molecule multiplex fluorescence in situ hybridization (smFISH) assays, we used unstained sections immediately adjacent (within 10 *μ*m) to the Visium sections. Description of the specimens used for spatial validation are available in Supplementary Data 5.

### Overview of the multi-omics approach

**Fig. 1:**
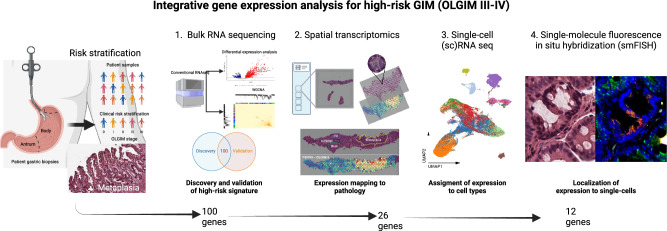
Overview of study design. Multi-omics flow diagram demonstrating process of discovering and orthogonally validating gene marker panel. At each step, the number of marker genes is shown. This figure was created with BioRender.com.

An overview of the multi-omics pipeline is provided in Fig. 1. In brief, this analysis included the following: (i) discovery of a high-risk gene expression signature using RNA-seq data (*N* = 88 samples; GAPS); (ii) validation of the high-risk genes in a held-out cohort using RNA-seq data (*N* = 215 samples; GAPS); (iii) mapping of the high-risk genes to metaplastic foci using a spatial transcriptomics assay (*N* = 5 samples; spatial cohort); (iv) determining of the overlap of the high-risk GIM spatial signature with differentially expressed genes in intestinal-type GC samples with RNA-seq data (*N* = 198 samples; TCGA); (v) assigning the high-risk, spatially mapped genes to specific cell subpopulations using single cell RNA-seq (scRNA-seq) (*N* = 40 samples; scRNA-seq cohort); and (vi) validation of a subset of these genes at single cell resolution using smFISH (*N* = 5 samples; spatial cohort). Genes selected at each step are outlined in Supplementary Data 6–9.

### Gene expression analysis of high- versus low-risk GIM

For the GAPS-based marker discovery phase, a detailed summary of the cohort’s demographic, clinical and histologic characteristics are provided in Table 1. The cohort was enriched for Asians (43%) and Hispanics (24%). When assessing OLGIM stages, 56.4% were OLGIM stage 0 (no GIM), 16.6% OLGIM I, 13.5% OLGIM II, 9.2% OLGIM III, and 4.2% OLGIM IV.

**Table 1:** Clinical and histopathologic attributes of GAPS cohort

Enrolled subjects (*N* = 163)		Unique gastric biopsies (*N* = 303)	
Characteristic	Frequency (%)	Finding	Frequency (%)
Age		All biopsies	
<50	40 (24.5)	Normal/NAG	204 (67.3)
50–69	90 (55.2)	CAG	99 (32.7)
>70	33 (20.2)	GIM severity	96 (31.7)
Female	105 (63.1)	Mild	42 (13.9)
Race/Ethnicity		Moderate	31 (10.2)
Non-Hispanic White	37 (22.7)	Severe	23 (7.6)
Black	1 (0.6)	Antrum	(*N* = 153)
Hispanic	39 (23.9)	Normal/NAG	92 (60.7)
East Asian	70 (42.9)	CAG	61 (39.9)
Other	16 (9.8)	GIM severity	60 (39.2)
Foreign born	102 (62.6)	Mild	26 (17.0)
Family history*	16 (9.8)	Moderate	20 (13.1)
Proton pump inhibitor use	53 (20.4)	Severe	14 (9.2)
OLGIM stage**		Body	(*N* = 150)
0 (no GIM)	92 (56.4)	Normal/NAG	112 (74.7)
I (lowest)	27 (16.6)	CAG	38 (25.3)
II	22 (13.5)	GIM severity	36 (24.0)
III	15 (9.2)	Mild	16 (10.7)
IV (highest)	7 (4.2)	Moderate	11 (7.3)
		Severe	9 (6.0)

Table 1 represents prospectively-recruited patients through GAPS (GAstric Precancerous conditions Study); clinical information on samples drawn from publicly-available data sources (e.g., TCGA cohort), as well as cancer resection specimens are not included in Table. *Family history defined as a first-degree relative diagnosed with gastric adenocarcinoma. **Gastric intestinal metaplasia (GIM) severity scores used to calculate operative link (OLGIM) stage. NAG, non-atrophic gastritis; CAG, chronic atrophic gastritis.

We used conventional RNA-seq to analyze 303 gastric specimens (153 antrum, 150 body) originating from 163 unique individuals from GAPS (specimen-level data on histopathology and sequencing metrics are provided in Supplementary Data 2). These samples were obtained at the same time point. Samples were stratified as high-risk if OLGIM stage was III or IV or operationally defined as low-risk if OLGIM < III. The cohort was separated into a discovery set of 88 samples (22 high-risk, 66 low-risk) from 46 patients and a held-out validation set of 215 samples (22 high-risk, 193 low-risk) from 115 patients. The discovery and validation sets were balanced with regards to the number of high-risk specimens; moreover, both sets had equal distribution of antral and body samples. Prior to differential expression analysis, we performed unsupervised clustering through (Supplementary Fig. 1) to confirm preferential grouping of high-risk and low-risk samples. Subsequently, we conducted differential expression analysis with limma-voom^17^, utilizing a factorial design strategy (Supplementary Fig. 2).

### Discovery of genes associated with high-risk GIM

From the discovery set, we identified a preliminary list of 399 genes that were differentially expressed in the high-risk samples (Supplementary Fig. 3A–C**;** Supplementary Data 6). Likewise, we excluded any genes which differential expression profile differed significantly between antrum and body (Supplementary Fig. 3D). Next, we conducted weighted gene co-expression network analysis (WGCNA)^18^ to determine groups of co-expressed genes, otherwise referred to as gene modules. Using hierarchical clustering with Pearson correlation distance, we demonstrated that genes from two modules were informative of high-risk gastric cancer precursors, whereas five other modules were not informative (Supplementary Fig. 4).

**Fig. 2:**
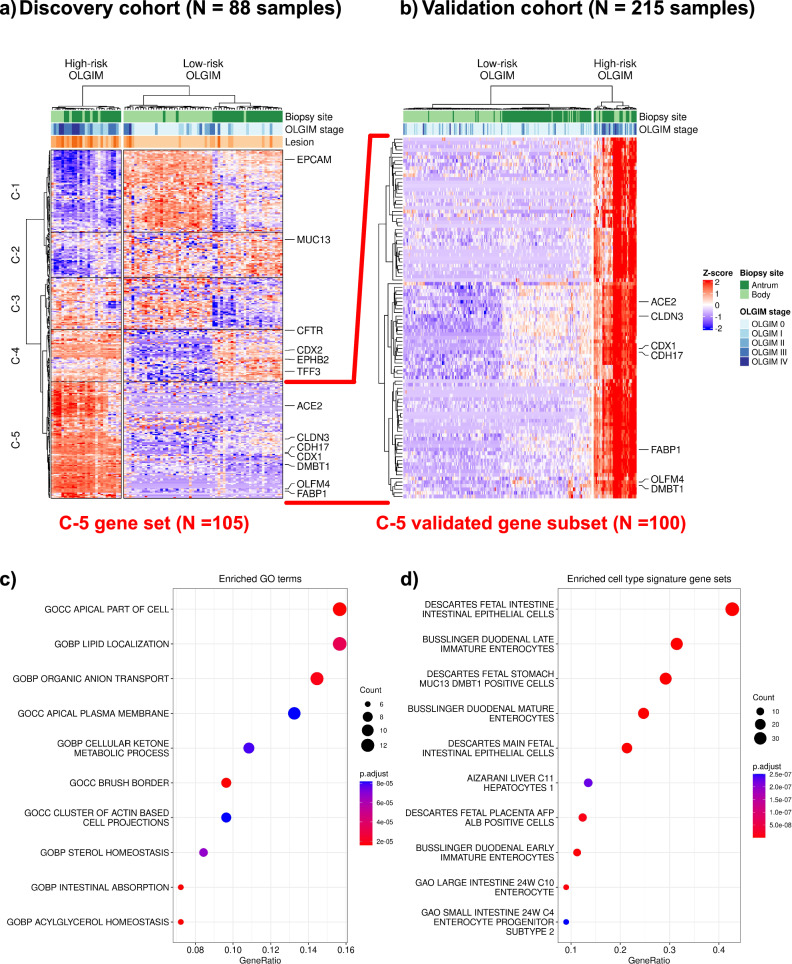
Discovery and validation of the high-risk expression signature. **a** Heatmap and hierarchical clustering of differentially expressed and co-expressed genes from the discovery cohort of 88 samples, 22 high-risk (defined as operative link stages III-IV) and 66 low-risk (defined as operative link stages 0-II). Most of the high-risk samples clustered distinctly and separately from the low-risk group, regardless of the anatomic site of the biopsy (top dendrogram). A set of genes were found to be both differentially expressed between high- and low-risk samples. Cluster-5 (C-5) represents 105 genes which were selectively upregulated in their expression only in high-risk samples, regardless of anatomic location. **b** We found 100 genes from C-5 to be differentially upregulated in the validation cohort (22 high-risk and 193 low-risk samples), confirming a robust signature for high-risk GIM which is agnostic of location. Dot plot depicting over-representation analysis results of these 100 genes: (**c**) gene ontology terms are enriched with intestinal processes (e.g., brush border, intestinal absorption); (**d**) cell type signature gene sets are enriched for mature and immature/fetal intestinal cell types.

We intersected genes from the two informative WGCNA modules and the differential expression analysis, resulting in a refined set of 314 genes that were: (i) differentially expressed in high-risk samples from both anatomic regions, and (ii) co-expressed in gene modules associated with high-risk stages (Fig. 2a, Supplementary Data 6). From this subset of 314 genes, we identified five discrete expression clusters that were labeled C-1 through C-5 (*side dendrogram*). Cluster C-5 represented a subset of 105 genes which were overexpressed in high- compared to low-risk samples, with the highest Z-scores. This gene set included established GIM markers such as *CDX1*, *FABP1* and *ACE2*. In addition to markers of mature enterocytes (*ANPEP*, *CDH17*)^19,20^, we also found markers of intestinal stem cells (such as *OLFM4*)^21^, and other immature intestinal lineages such as transit-amplifying cells (*DMBT1*)^22^. We termed the population of immature cells found in the gastric epithelium expressing intestinal stem cell markers as “intestinal-like stem cells”.

### Validating the genes associated with high-risk GIM

Next, we validated these results in the independent validation set of 215 samples. For the 105 genes identified from the C-5 cluster in the discovery set, we found a striking 100 out of 105 genes (95.2%) consistently overexpressed among the validation set’s high-risk samples (Fig. 2b). The full gene list is provided in Supplementary Data 7. To characterize the functional pathways and cellular associations of these 100 genes, we conducted over-representation analyses with clusterProfiler^23,24^. We selected gene sets relative to gene ontology and cell types from the MSigDB database^25–27^. Enriched gene ontology terms (Fig. 2c) included intestinal absorption (*SLC2A5*, *ABCG8*, *ABCG5*, *MOGAT2*, *PRAP1*, *FABP1*) and presence of a brush border (*ACE2*, *SLC28A1*, *SLC2A2*, *MME*, *SLC6A19*, *SLC7A9*, *MTTP*, *MYO7B*) among other intestinal-related processes. Consistent with these findings, we found enrichment of certain cell lineage gene sets (Fig. 2d) including mature (*SLC2A5*, *APOC3*, *ACE2*) and fetal enterocytes (*LRRC19*, *CELP*, *RBP2*), as well as immature enterocytes (*DMBT1*, *CPS1*). A comprehensive listing of enriched gene ontology terms and cell lineage gene sets are available in Supplementary Data 10, 11.

### Spatial transcriptomics maps the high-risk expression signature to metaplastic foci

**Fig. 3:**
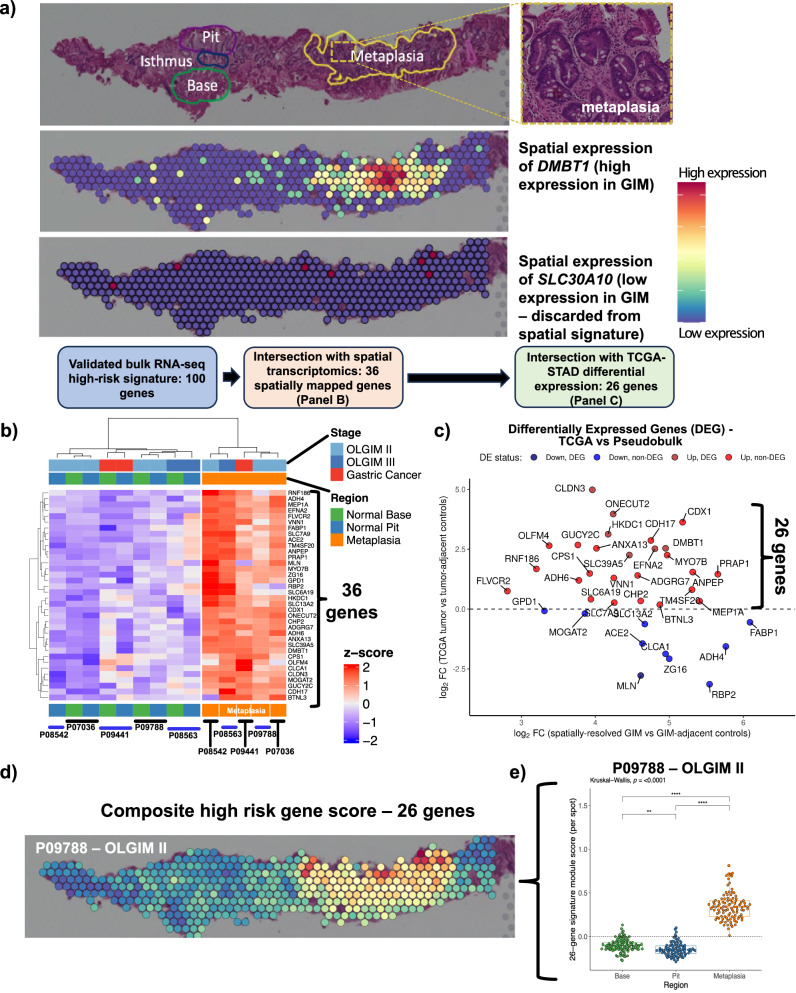
Spatial resolution of the high-risk signature. **a** An example of the expression profile of *DMBT1* upon a Visium slide annotated by a pathologist for areas of normal glandular architecture (base and pit) and metaplasia. *DMBT1* is shown as an example of a spatially resolved gene mapping to pathologist-annotated metaplasia, whereas *SLC30A10* is shown as an example of a gene not mapping to metaplasia and, thus, discarded form the spatially-resolved signature. **b** Heatmap depicting 36 differentially expressed genes from spatial pseudobulk analysis that overlapped with bulk RNA-seq signature from GAPS (FDR-adjusted *P* value ≤ 0.05; analysis performed using limma-voom). **c** Scatter plot showing log2 fold-change of 36 upregulated genes from the spatial cohort (X-axis) and log2 fold-change from TCGA. Twenty-six genes overexpressed in both analyses are shown in red. **d** Spatial mapping of the refined 26-gene signature onto Visium spots. **e** Comparison of 26-gene signature between metaplastic foci vs normal stomach base or pit (Kruskal-Wallis and Dunn test FDR-adjusted *p* < 0.001). Note: each Visium spot is 55 µm in diameter, with 100 µm distance between the center of adjacent spots.

Next, we used a spatial expression assay (Visium, 10X Genomics) to map the genes of the high-risk expression signature to GIM regions. We generated extensive histopathology annotation for these samples, all which included regions of (i) normal base, (ii) normal pit or (iii) metaplastic foci. An example of one GIM sample (**P09788**) is shown in Fig. 3. The aggregated spots per region for each sample were used to conduct a differential expression analysis comparing regions of metaplasia vs normal pit or base, using a ‘pseudo-bulk’ analysis. Significantly upregulated genes were defined as those with a positive fold-change and an FDR-adjusted *P* value ≤ 0.05. Among all samples, we determined that 458 genes were significantly upregulated in regions of metaplastic foci compared to both normal gland base and pit (Supplementary Data 8).

Next, we intersected the 100 genes from the validated expression signature as previously described with the 458 genes that were mapped using the spatial transcriptomic assay. Notably, from the validated expression signature, 36 out of 100 (36%) genes were expressed specifically in regions of the metaplastic glands (e.g., *DMBT1*, Fig. 3a; other spatially resolved genes in Fig. 3b). Overall, this result identified a subset of 36 high-risk differentially expressed genes that mapped to pathologist-annotated regions of metaplasia.

### The high-risk expression, spatially mapped signature’s association with gastric cancer

We determined how many of the 36 spatially mapped high-risk genes were also differentially expressed in the intestinal subtype of GC. This step of the analysis used RNA-seq data from the TCGA-STAD cohort. We conducted differential gene expression analysis between 180 gastric cancers, all being of the intestinal subtype, and 18 matched tumor-adjacent gastric tissues. We compared the fold-change from the TCGA analysis vs the fold-change from the spatial gene expression analysis for the 36 genes (Supplementary Data 9). Twenty-six genes overlapped between those which were significantly overexpressed in high-risk GIM (relative to low-risk GIM), localized to metaplastic foci, and were consistently upregulated in GC (Fig. 3c).

We quantified the expression of these 26 genes in gastric metaplastic foci using a composite signature score^28,29^. The 26-gene score was significantly higher among the Visium spots mapping to metaplastic foci for each of the five spatial samples compared to areas with normal stomach base or pit (Kruskal-Wallis and Dunn test FDR-adjusted *p* < 0.001) (Fig. 3d and Supplementary Fig. 5). Overall, this set was highly specific for metaplasia and did not map to any other normal gastric regions. The gene signature included established markers for immature intestinal lineages (*OLFM4*, *DMBT1*)^21,22^ and markers for mature enterocytes (*ANPEP*, *CDH17*)^19,20^.

### The high-risk spatial signature is expressed in both enterocytes and intestinal-like stem cells

We used scRNA-seq to determine the 26 gene signature across all cells in the 40 specimens of GC, precancerous lesions, and normal samples. The joint data set contained a total of 116,643 single cells. From this data set we identified nine major cell lineages: epithelial, T cells and NK cells, B cells, stromal cells (fibroblasts), plasma cells, endothelial, myeloid (macrophages and dendritic cells), mast cells, and smooth muscle cells. This signature was highly specific to the subset of epithelial cells (Supplementary Fig. 6 and Supplementary Data 12).

**Fig. 4:**
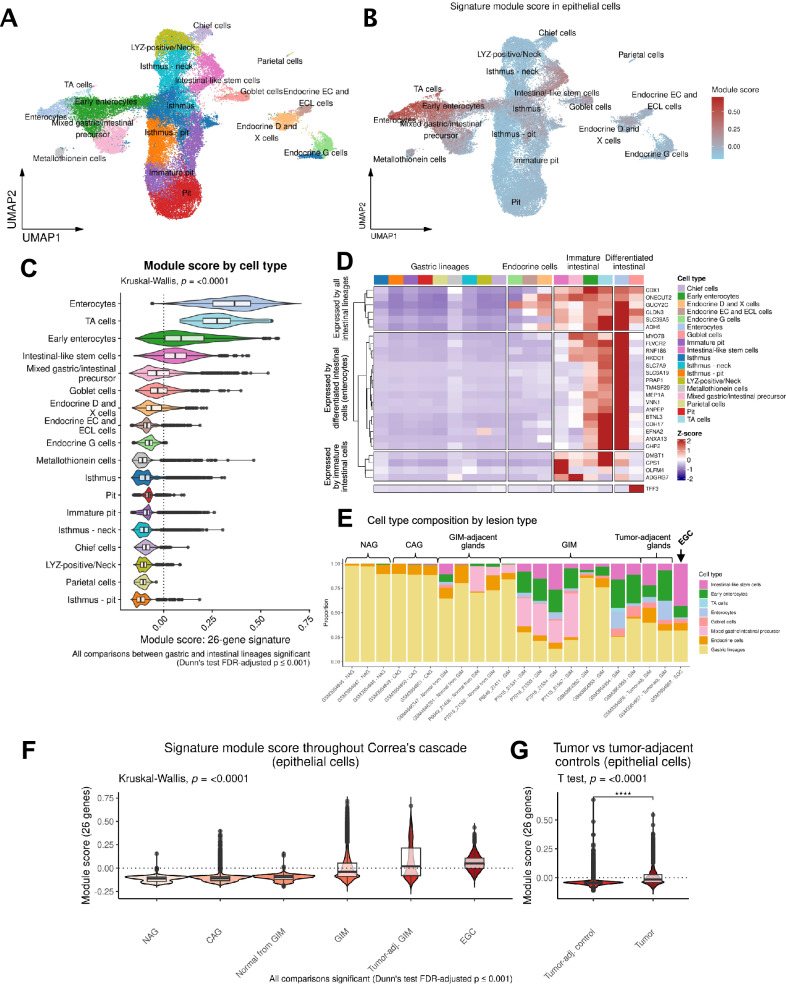
Single-cell identification of cell types expressing the high-risk signature. **A** Uniform Manifold Approximation and Projection (UMAP) plot showing reference-mapped epithelial cells. **B** UMAP plot showing module score by epithelial cell type. **C** Comparison of the module score between cell types using all 40 samples from the scRNA-seq cohort. TA, transit amplifying cells. **D** Heatmap showing the scaled expression of the 26 genes by cell type. **E** Stacked bar plots depicting the proportion of cell types per sample, ordered by stage of Correa’s cascade. Gastric lineages are aggregated into a single class. EGC Early gastric cancer. **F** Comparison of module score across Correa’s cascade (*p* < 0.001 for all comparisons). **G** Comparison of the module score between GC and tumor-adjacent control tissues (*p* < 0.0001).

We next analyzed the epithelial cell subset at the single-cell level. We annotated 18 cell clusters based on expression of specific markers (Fig. 4A and Supplementary Fig. 7). We first identified broadly gastric (*TFF2*, *MUC5AC*) and broadly intestinal (*REG4*) cells. The gastric cell clusters included chief cells (*LIPF*, *PGA3*, *PGA4*, *PGA5*), parietal cells (*ATP4A*, *ATP4B*, *GIF*, *CKB*), isthmus cells (*STMN1*, *MKI67*, *HMGB2*), pit cells (*CAPN8*, *TFF1*, *GKN1*, *GKN2*, *SULT1C2*), endocrine G cells (*NKX6-3*, *GAST*, *CHGB*), LYZ-positive/neck cells (*AQP5*, *MUC6*, *PRR4*, *LYZ*), and metallothionein-expressing cells (*MT1X*, *MT2A*, *MT1G*, *MT1H*, *MT1E*).

Among REG4^+^ intestinal lineages, we identified clusters of early and mature enterocytes (*CDH17*, *FABP1*, *FABP2*, *KRT20*, *GPA33*), goblet cells (*SPINK4*, *MUC2*, *TFF3*), transit-amplifying cells (*DMBT1*), and intestinal-like stem cells (*OLFM4*, *LEFTY1*). Intestinal-like stem cells shared some common transcriptional features with isthmus cells, including expression of *MKI67*, *STMN1* and *HMGB2*. Roughly 10% of intestinal-like stem cells expressed *CDCA7*, similar to what was observed in LYZ-positive/neck cells. Interestingly, a small fraction of these cells (~5%) also expressed *LGR5*, which was again found with a similar percentage among LYZ-positive/neck cells. We collectively termed immature cells with intestinal features (mixed gastric/intestinal precursors, early enterocytes, transit amplifying cells, and intestinal-like stem cells) as “immature intestinal cells” for the purposes of further analysis. We found distinct sets of genes from the 26-gene signature to be significantly enriched in mature enterocytes compared to immature intestinal cells (Fig. 4B, C, Supplementary Data 13). Notably, the 26-gene signature was nearly absent among all normal gastric lineages.

Next, we examined the expression of each gene scaled across the epithelial cell types to highlight which cells displayed the highest levels of expression. As a specific marker for goblet cells was not part of the original signature, we also included the gene *TFF3*^30,31^. The gene dendrogram revealed three distinct groups of genes; six genes expressed by all intestinal lineages (*ADH6, SLC39A5, GUCY2C, CLDN3, ONECUT2*, and *CDX1*), 16 genes expressed by only mature intestinal lineages (*MYO7B, FLVCR2, RNF186, HKDC1, SLC7A9, SLC6A19, PRAP1, TM4SF20, MEP1A, VNN1, ANPEP, BTNL3, CDH17, EFNA2, ANXA13*, and *CHP2*), and four genes expressed preferentially by immature intestinal cells (*OLFM4*, *ADGRG7, CPS1*, and *DMBT1*) (Fig. 4D). Some genes like *CPS1*, despite being expressed predominantly by intestinal-like stem cells, were also expressed (at lower levels) among differentiated enterocytes. Similarly, some genes (such as *HKDC1*) showed strongest expression levels in differentiated enterocytes but were also expressed to a lesser degree among intestinal-like stem cells.

We analyzed the proportion of different cell lineages throughout Correa’s cascade (Fig. 4E). Normal, NAG, and CAG gastric tissue samples were mostly devoid of intestinal cells. In contrast, GIM was characterized by strong presence of both mature and immature intestinal lineages. Interestingly, GC was characterized by a significant enrichment of intestinal-like stem cells and substantial relative loss of differentiated goblet cells and enterocytes. These results suggest that the continued expansion of immature intestinal populations in GIM may be an important indicator and contributor to progression of intestinal-type GC. Next, we observed that the 26-gene signature score increased with the progressive stages of Correa’s cascade and in GC compared with tumor-adjacent controls (Supplementary Data 14, Supplementary Data 15, and Fig. 4F). Notably, there was a significant increase in the signature module score in tumor-adjacent GIMs compared to GIM from non-cancer patients. In a separate analysis, we analyzed the signature score between GCs and patient-matched tumor adjacent control tissues from a previous publication from our group^32^ (Fig. 4G). As expected, we found the 26-gene signature to be significantly increased in tumor cells relative to tumor-adjacent gastric tissue (Welch’s *T* test *P* < 0.0001).

### Single-molecule fluorescent in situ hybridization reveals intestinal-like stem cells in the isthmic/crypt region of metaplastic glands

The smFISH assay uses in situ RNA hybridization to visualize the spatial expression of up to twelve genes at single-molecule subcellular resolution and enables simultaneous incorporation of spatial and cellular-level gene expression data. Based on the scRNA-seq results that defined the aberrant intestinal-like stem cell populations, we selected eleven of the signature genes for smFISH. These genes included markers for immature intestinal cells (*OLFM4, CPS1, DMBT1)*, enterocytes (*HKDC1, ANPEP, CDH17, CLDN3, ANXA13*), or were expressed across all intestinal lineages (*CDX1, SLC39A5, ONECUT2*). We also included *TFF3* as a specific goblet cell marker. After imaging, the results were compared to the matching H&E images with pathology interpretation.

**Fig. 5:**
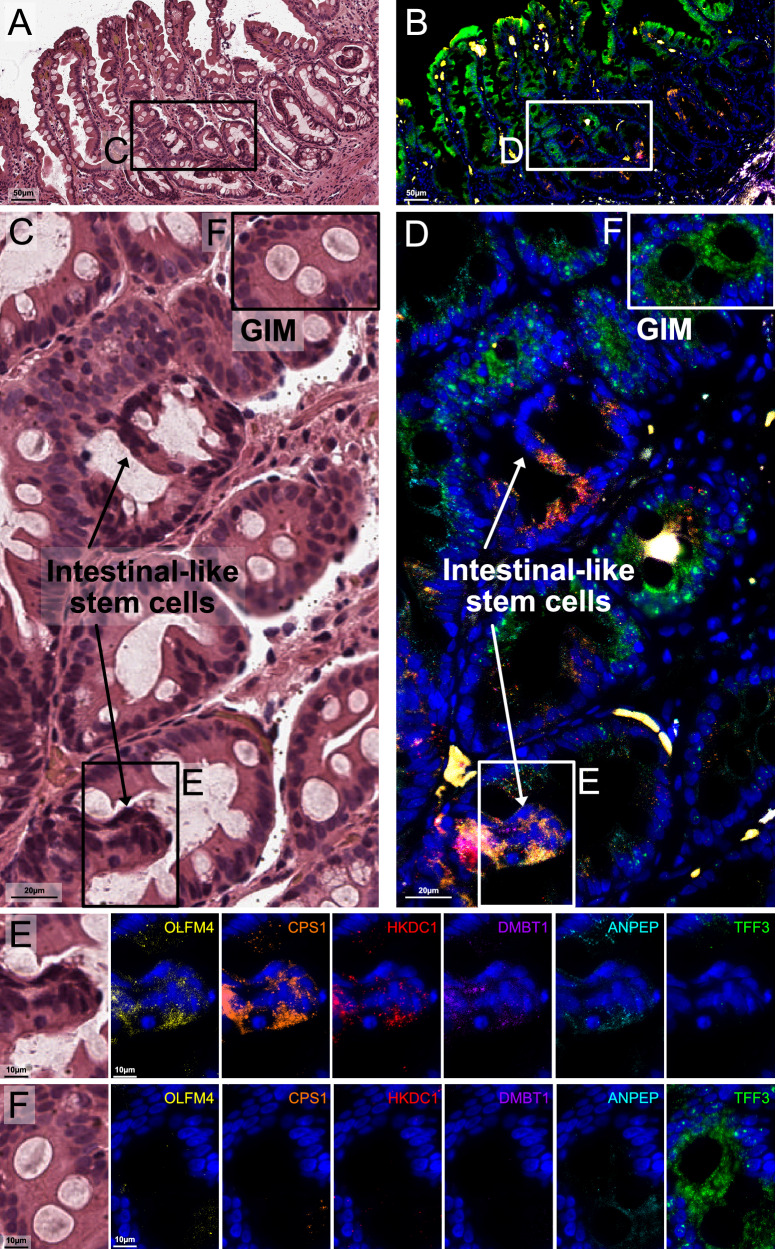
Single-molecule fluorescence in situ hybridization (smFISH) of gastric intestinal metaplasia (GIM). **A**, **B** Representative region showing H&E staining of GIM from sample P08563 (operative link III), and superimposed smFISH characterization for six genes: *OLFM4, CPS1, HKDC1, DMBT1, ANPEP*, and *TFF3*. **C**, **D** Inset magnification for highlighted area from (**A**) and (**B**), respectively (90-degree clockwise rotation). **E** Highlighted regions in (**C**) and (**D**), showing H&E and individual channels from areas enriched for intestinal-like stem cells. These cells show elevated expression of *OLFM4, DMBT1, CPS1*, and *HKDC1*, together with moderate expression of *ANPEP*. **F** Highlighted regions in (**C**) and (**D**), showing H&E and individual channels from areas enriched for well-differentiated (mature) cells. These cells show elevated expression of *TFF3* and moderate expression of *ANPEP*. The expression of these genes was mutually exclusive, in space, from the immature cell markers.

Notably, none of the selected genes were expressed in the normal gastric glands across any of the samples. We identified several distinct cellular compartments which occurred solely within metaplastic tissue (Fig. 5, Supplementary Figs. 8–10). The first compartment consisted of mature or differentiated intestinal cells and were characterized by strong expression of *TFF3* (goblet cells) and moderate *ANPEP* signal (enterocytes). A second compartment consisted of cells which strongly expressed stem markers (O*LFM4, DMBT1* and *CPS1*); these columnar cells were characterized by high nuclear-to-cytoplasm ratio, were located near crypt regions of metaplastic glands, and were mutually exclusive in space to the mature markers. These results provide additional evidence confirming the presence of intestinal-like stem cells as previously identified in the scRNA-seq results. There were some genes (*ONECUT2* and *HDKC1*) which were ubiquitously expressed in both mature and immature cells. However, their expression was notably higher among the O*LFM4, DMBT1* and *CPS1*-positive stem cells at the gland isthmic/crypt region. In one early GC sample, we observed that the expression levels of immature intestinal cell markers (*OLFM4*, *DMBT1*, and *CPS1*) along with *CDX1* and *HKDC1*, were significantly higher in a region containing poorly differentiated tumor glands compared to an area with well-differentiated tumor glands, which prominently expressed *TFF3* (Supplementary Fig. 11). These findings align with the cell phenotypes seen in H&E staining and reflect the molecular characteristics of both intestinal-like stem cell regions and the differentiated goblet and enterocyte areas observed in GIM. The results from smFISH provided single-cell spatial resolution and confirmed the presence of distinct cellular compartments within metaplastic glands consisting of either mature intestinal lineages (enterocytes and goblet cells) or immature lineages.

## Discussion

In this study, we analyzed a cohort of individuals with pathology across the gastric precancerous spectrum. Using integrated bulk gene expression, spatial transcriptomics, scRNA-seq, and smFISH, we identified a highly refined signature of 26 genes which characterizes both the presence and cellularity of metaplasia within gastric glands. Notably, this set of genes is expressed by aberrant epithelial cells not typically found in healthy gastric tissue, reflects the presence of both mature intestinal lineages, such as goblet cells and enterocytes, and immature intestinal lineages, including intestinal-like stem cells within the metaplastic foci. Furthermore, this discrete gene set holds potential for distinguishing between these mature and immature aberrant lineages.

While this high-risk gene expression signature is characterized by both markers of mature enterocytes and stem cells, we found that the more advanced lesions had greater expression of immature intestinal markers. This progression was characterized by increased expression of intestinal-like stem cell markers such as *OLFM4*^21^, as well as markers of transit-amplifying cells such as *DMBT1*^22^. These markers were absent from both normal differentiated gastric tissues, as well as gastric stem cells. Collectively, these results point to immature intestinal cells playing an important, constitutive role in the biology of advanced preneoplasia. A recent cohort study of a high-risk Chinese Singaporean population followed patients with GIM to neoplasia, and analyzed GIM tissue using a combination of single-cell and spatial analyses^33^. This study similarly found GIM tissue to consist of enterocyte-dominant and stem-cell-dominant regions, with expansion of the stem-cell-dominant compartment linked to early malignancy. Our study importantly complements and validates the Singaporean study by offering a similar biological conclusion in a vastly different population; at the same time, our study offers further spatial refinement on the location and origin of these intestinal-like stem cell compartments through smFISH. In addition, another recent report demonstrated phenotypic mosaicism in GIM, by which individual GIM cells co-express both intestinal and gastric markers^34^. This interesting finding strongly suggests that the intestinal cells identified by our studies also possess gastric transcriptional properties. Future efforts should be made to fully characterize these cell populations. Our data lend further credence to the hypothesis that terminally-differentiated epithelial cells (such as enterocytes and goblet cells) may simply be passive bystanders harboring genetic alterations already present in genetically unstable stem cells, the latter of which have the potential to become the true carcinogenic precursors^35^.

Among the genes from the high-risk signature, some correspond to known markers for immature intestinal cells, including *OLFM4* and *DMBT1*. Other genes in the signature are established markers for mature enterocytes. For example, *ANPEP* encodes aminopeptidase N, and an early report suggested that leucine aminopeptidase activity was highly specific to metaplastic zones within the human stomach examined microscopically^19^. *CDH17* is a membrane-associated enterocyte marker that has been found expressed in >60% of GCs, with greater expression specifically in intestinal-type GCs^20^. In our study, we found that *CPS1* to localize mostly (though not exclusively) to immature intestinal compartments. Interestingly, *CPS1* is an enzyme of the urea cycle and has previously been associated with GIM^36^. Thus far, *CPS1* has not been associated with stem cell biology. However, a recent report in lung cancer suggests that *CPS1* may be crucial for pyrimidine maintenance and DNA synthesis in *KRAS* mutant cells^37^. Notably, *KRAS* was one of the driver oncogenes previously identified^33^, suggesting that there may be a relevant and novel role for *CPS1* as a source supply of pyrimidines in the context of DNA synthesis among replicating precancerous cells.

*HKDC1* is a hexokinase and has not been previously described in gastric preneoplasia. In vitro studies suggest *HKDC1* may promote chemoresistance, proliferation, and epithelial-to-mesenchymal transition of gastric cells through induction of NF-κB^38^, and that *HKDC1* may be pivotal for glycolysis and proliferation in GC cells^39^. *HKDC1* has been found to promote lung^40^, breast^41^, and biliary^42^ cancers. Moreover, deletion of *HKDC1* inhibits proliferation and tumorigenesis in mice^43^. Our findings suggest *HKDC1* may be a novel marker for advanced gastric preneoplasia. Spatial co-expression of *HKDC1* and *CPS1* with *CDX1, OLFM4* and *DMBT1* in GIM samples, as well as GC, provide strong evidence of the potential role of these cells in metabolic reprogramming of GC precursor lesions.

Most prior molecular and genomic studies of the GIM microenvironment have focused on populations with moderate-to-high Hp prevalence and high GC incidence^33,44,45^. Our study addresses a gap in the literature by providing needed mechanistic data on advanced GIM in a relatively low-risk population common to many regions of North America and Europe. An additional motivation for selecting Hp-negative patients for cohort development is that almost all patients diagnosed with GIM in developed nations have already undergone Hp eradication therapy—that is to say, by the time such patients come to clinical attention they have cleared infection. There currently is no reliable test to distinguish between individuals with eradicated prior infection and no prior infection, as Hp antibody titers fall substantially in the years following clearance^46^. The ongoing carcinogenic potential of Hp-negative GIM may in part be explained to the establishment of clonal stem cell lineages, as suggested by this and other studies^33,45^.

Our findings have public health significance. As only a small fraction of patients with GIM will progress to GC over long-term follow-up^6,7,47–49^, indefinite endoscopic surveillance of these patients may lead to unnecessary cost, medical risk, and anxiety. OLGIM score has been found to be significantly associated with cancer or dysplasia progression risk^11,12^. However, the widescale adoption of OLGIM may be limited by the high interobserver variability and manually-intensive nature of its histologic calculation. The signature highlighted in this study may aid in this classification, by providing a molecular correlate to metaplastic cellularity and extent. Our study also offers molecular insight into the histologic subtyping of GIM into complete and incomplete phenotypes. One recent study using both mouse models and human participants demonstrated *ANPEP* expression to be strongly associated with incomplete GIM^50^. Another study found *OLFM4* to be a key marker overexpressed in human incomplete GIM samples, and that this overexpression promoted tumor-like behaviors through Wnt/β-catenin signaling^51^. Our study extends these findings by offering spatial context to the expression of both markers to immature compartments within GIM. Moreover, our results may help to explain the increased GC risk faced by individuals harboring incomplete GIM mediated through enrichment of intestinal-like stem cells.

Our study has strengths and limitations. Our study benefited from the prospectively collected specimens (GAPS) which contained detailed histologic severity scoring (OLGIM) which is not commonly available. By contrast, public data sets of GIM were available only in broader classes of Correa’s cascade (e.g., NAG vs CAG vs GIM). Notably however, these less-phenotyped public data sets were only used for marker refinement. Our spatial cohort was limited in size. We note that spatial validation assays are newly-emerging approaches, and that each sample in the validation steps required detailed annotation by an expert pathologist. As such, each slide represents hundreds of independent, phenotyped data points on which analysis was performed. Our study was cross-sectional in nature and did not contain longitudinal data on GIM progression. The motivation of our study was not to develop a risk-prediction model, but rather to understand molecular and cellular differences between high- and low-risk lesions. Our future studies will be focused on establishing causal inference through prospective longitudinal cohorts.

Leveraging multiple independent cohorts, we utilized integrated transcriptomic approaches incorporating both spatial and single-cell methods to further characterize the molecular and cellular characteristics of high-risk GIM. We identified a discrete set of 26 genes which are associated with advanced OLGIM stages, localize spatially to metaplastic foci, are expressed by aberrant epithelial cells, are differentially expressed in intestinal-type GC, and can be used to distinguish mature and immature intestinal cells in GC precursors. We find that with increasing histologic severity, the expression of intestinal-like stem cell markers increase. These data hold important future implications for future cancer interception.

## Methods

### Research ethics

This research involved human subjects, and was performed in accordance with the Declaration of Helsinki. The human subjects research was approved by the Stanford University Institutional Review Board (approval number 45077). Informed consent to participate was obtained from all participants.

### Standard RNA-seq of gastric tissue samples

The gastric tissue was processed using the TissueLyser LT compact bead mill (Qiagen, Venlo, Netherlands) per manufacturer’s protocol using 5 mm stainless steel beads in 600 µL lysis buffer. AllPrep® DNA/RNA/miRNA Universal (Qiagen) spin column was used to bind the DNA, and RNA was extracted from the flowthrough was then used to extract the per manufacturer’s protocol. RNA quality was assessed using Qubit RNA Broad Range Assay Kit and Qubit Fluorometer system (Thermo-Fisher Scientific, Massachusetts, USA). To assess the quality of the RNA, the samples were analyzed using the LabChip GX system (RNA Assay—Standard Sensitivity Perkin Elmer) as per the manufacturer’s protocol. RNA samples with an RNA quality score (RIN value) greater than 4 were then used for direct DNA library generation using the KAPA mRNA HyperPrep Library Preparation Kit (Roche). TruSeq DNA UD Indexes (Illumina) were used for adapter ligation. The KAPA Library Quantification Kit (Roche) was used for library quantification. Library quality was assessed using an iSeq 100 system and the i1 Reagent v2 (Illumina). Sequencing was conducted on an Illumina Novaseq.

### Bulk RNA-seq analysis (GAPS cohort)

RNA sequencing reads were aligned to GRCh38 using STAR aligner version 2.7.10b. Counts per gene were generated using --quantmode GeneCounts option with STAR. We split the RNA-seq data into 2 independent sets: a discovery cohort comprising 88 paired biopsies from 46 individuals (46 antrum, 42 body; 22 high-risk OLGIM, 66 low-risk OLGIM) and a validation cohort comprising 115 individuals (215 paired patient biopsies: 115 antrum, 100 body; 22 high-risk OLGIM, 193 low-risk OLGIM).

We used filterByExpr to filter out lowly-expressed genes followed by TMM normalization method from edgeR package in the R statistical programming environment^52,53^. Next, we performed unsupervised clustering through hierarchical clustering and principal components analysis on both the discovery and validation cohorts to confirm preferential grouping of samples into high- and low-risk OLGIM groups (Supplementary Fig. 1A–D).

A schematic of the data analysis pipeline for the bulk RNAseq data is shown in Supplementary Fig. 2. For both the discovery and validation data sets, we performed differential expression analysis in the same fashion. Specifically, we utilized a factorial design strategy to compare high- and low-risk lesions for each anatomic region (body and antrum). In addition, most samples had matched patient biopsies from both antrum and body: for the discovery cohort this involved 42/46 samples (91.3%) and for the validation cohort this involved 100/115 samples (87%). We used voom and duplicateCorrelation from limma^17^ to estimate patient-specific weights for the regression fit, and a fold-change threshold of 1.25 was used to identify differentially expressed genes. To adjust for batch effects in the validation data set, comprised of 3 batches, we incorporated the sequencing batch as a co-variable for the regression models. Significance was set at 0.05 (FDR-adjusted P-values). In addition, we calculated the significance of the double variable (interaction term between the risk strata and anatomic region) over the differential expression profiles.

WGCNA was conducted using WGCNA R package^18^. The top 15% most variable genes (*N* = 5797) were used as input for WGCNA and normalized using the VST method in DESeq2 R package^54^. A soft threshold of 16 was selected for the generation of signed weighted networks, to achieve a scale-free topology model fit R^2^ > 0.8 and a mean connectivity < 100. Informative gene modules were identified based on module-trait relationship and hierarchical clustering (Supplementary Fig. 4). The differentially expressed genes were intersected with genes from informative modules from WGCNA to identify a set of genes both (i) significantly upregulated and (ii) co-expressed, common to the body and antrum of the stomach (Supplementary Fig. 4D). A total of 314 genes met these criteria. We conducted hierarchical clustering using the scaled expression levels of these 314 genes with ComplexHeatmap^55^ in R. We identified 5 gene clusters defining high-risk GIM. We selected a subset of 105 genes from a distinct cluster with the highest Z-score (C-5) for further validation in the held-out testing set (Fig. 2a).

### Validation of the high-risk expression signature

To validate the high-risk gene expression signature, we performed differential expression analysis as described above, in the held-out testing set. Differentially expressed genes common to the body and antrum of the stomach were intersected with common differentially expressed and co-expressed genes from the body and antrum in the discovery cohort. This yielded 100/105 (95.23%) overlap between the discovery and validation sets. Functional annotation of these 100 genes was performed through over-representation analysis, with the enricher function in clusterProfiler^23,24^ R package. Gene sets were imported into R from Molecular Signature Database (MSigDB) using R package msigdbr.

### Spatial transcriptomics assay

The Visium Spatial for FFPE Human Transcriptome Gene Expression Kit Gene Expression Kit (version 1) (10X Genomics) was used to prepare libraries according to the manufacturer’s protocol. Briefly, 10 μm thick tissue sections from FFPE blocks were placed on a Visium Spatial Gene Expression slide, deparaffinized and stained with hematoxylin and eosin before coverslip removal and decrosslinking. Slides were imaged using a Keyence BZ-X microscope. Probe hybridization, ligation, release, extension, and library construction were performed as per protocol using 17 cycles for sample index PCR, and libraries were sequenced on an Illumina Novaseq 6000.

The Space Ranger (10x Genomics) version 1.3.1 mkfastq command was used to generate Fastq files, and Space Ranger version 1.3.1 count was used with default parameters and alignment to GRCh38 to perform image alignment, tissue detection, barcode and UMI counting, and generation of feature-barcode matrix. Tissue regions were annotated by a pathologist (author JS) on a hematoxylin and eosin histology images from the Visium slide.

Raw counts were imported into the Seurat R package (version 4.3.0) and low-quality spots that detected <500 genes were removed. Genes detected in 3 or fewer spots were excluded, and counts were normalized with SCTransform. Spots within anatomically distinct tissue regions were labeled by unsupervised clustering refined by pathologist annotations. Spots were first clustered by FindClusters (resolution = 1.4) on the first 20 principal components and clusters marking tissue regions were annotated by pathologist annotations. Tissue regions were defined by transferring pathologist annotations to Loupe Browser (10x Genomics) version 6.1.0 to relabel any spots that did not match the pathologist annotations. Raw counts were summed from spots from each region of each patient sample to generate a pseudobulk count matrix, which was preprocessed using edgeR TMM normalization method in R. The limma-voom strategy was utilized to determine differentially expressed genes between metaplasia and spots mapping to normal gland base and pit. Gene signature scoring for each spot was performed with the AddModuleScore function in Seurat. To compare the gene signature module scores between metaplasia and normal gland pit or base regions. Normality of the data was assessed using Shapiro-Wilk test, followed by Bartlett test for homogeneity of variance and Welch T tests or Kruskal-Wallis followed by Dunn test for group comparisons.

### RNA-seq analysis from the TCGA data

The differential expression analysis between tumor and adjacent non-tumor tissues from the TCGA RNA-seq dataset was performed to identify which of the spatially-resolved high-risk genes continued to be expressed at similar or increased patterns in cancerous tissue. We had multiple metrics and characteristics from these cancer specimens. RNA-seq data from 448 samples, including patient-matched tumor and non-tumor tissues, was downloaded and processed using GDCquery, GDCdownload and GDCprepare functions from TCGAbiolinks R package^16^. The functions assay and colData from R package SummarizedExperiment were used to extract raw count matrices and sample metadata. We subset the cohort to include only intestinal-type tumors and their patient-matched control tissues (n = 198; 180 tumor and 18 matched non-tumor samples) prior to filtering and normalization. Differential expression analysis was conducted using the limma-voom strategy. We used patient IDs as blocking variables for the regression models.

### Single-cell RNA sequencing

Tissue biopsies were dissociated using a combination of enzymatic and mechanical dissociation with a gentleMACS Octo Dissociator (Miltenyi Biotec), and the resulting cells were cryofrozen using 10% DMSO in 90% FBS (ThermoFisher Scientific, Waltham, MA) in a CoolCell freezing container (Larkspur, CA) at −80 °C for 24–72 h followed by storage in liquid nitrogen. The cells were rapidly thawed in a bead bath at 37 °C, washed twice in RPMI + 10% FBS, and filtered successively through 70 μm and 40 μm filters (Flowmi, Bel-Art SP Scienceware, Wayne, NJ), washed, filtered, and counted using 1:1 trypan blue dilution. Cells were concentrated between 500–1500 live cells/μl. The scRNA-seq libraries were generated using the Chromium Next GEM Single Cell 5’ version 2 protocol, targeting 10,000 cells with 14 PCR cycles for cDNA and library amplification. The sequencing was performed on an Illumina NovaSeq 6000.

We used Cell Ranger (10x Genomics) version 3.1.0 mkfastq command to generate Fastq files and count with default parameters aligned to GRCh38 to generate a matrix of unique molecular identifier (UMI) counts per gene and associated cell barcodes. Seurat (version 4.0.1)^28,29^ was used to construct Seurat objects from each sample. Quality control filters were applied to remove low-quality cells expressing fewer than 200 genes or greater than 30% mitochondrial genes, as well as doublets with UMI counts greater than 5000 or 8000. We removed genes that were detected in less than 3 cells. Data were normalized using SCTransform and first 20 principal components with a resolution of 0.6 or 0.8 were used for clustering. We removed computationally identified doublets from each dataset using DoubletFinder (version 2.0.3)^56^. The ‘pN’ value was set to default value of 0.25 as the proportion of artificial doublets and the ‘nExP’ was set to expected doublet rate according to Chromium Single Cell 3’ version 2 reagents kit user guide (10x Genomics). These parameters were used as input to the doubletFinder_v3 function with number of principal components set to 20 to identify doublet cells. Individual Seurat objects were merged and normalized using SCTransform. Downstream analyses requiring Seurat were conducted using version 4.4.0.

The data sets were integrated using a soft variant of k-means clustering implemented in the Harmony algorithm (version 0.1.0)^57^, using the RunHarmony function. This reduction was used in both RunUMAP and FindNeighbors functions for clustering. The first 20 principal components and a resolution of 1 were used for clustering. The data were normalized using SCTransform. The effects of variation in the mitochondrial gene percent were regressed out by in the SCTransform function. All further analyses with including differential expression analysis were conducted using data from the “SCT” assay. Major cell lineages were identified based on marker gene expression (Supplementary Fig. 6A). The Heatmap function from ComplexHeatmap^55^, FeaturePlot, DimPlot, and VlnPlot functions from Seurat were used for visualization. Dotplots for cluster marker visualization were generated with Clustered_DotPlot function from scCustomize (version 2.1.2). We performed a secondary clustering analysis of the epithelial cells with integration across samples using Harmony and a cluster resolution of 1.0.

Cell identity of the epithelial lineages was performed based on cell markers (Supplementary Fig. 7). Following label assignment, we refined our cluster labels using the output from SingleR algorithm^58^ as a confirmatory analysis. We used a single-cell atlas of normal stomach and duodenum as reference for automated annotation of cells^25^. Briefly, counts from the reference atlas were normalized to the logarithmic scale and used as a reference for automated annotation per cell using SingleR (version 1.14.1)^58^. Raw counts were used to annotate test datasets. Labels were predicted for each cell in the test dataset using the ‘SingleR’ function to calculate the Spearman correlation for marker genes for the reference dataset identified with Wilcoxon Rank Sum test. A second scRNAseq dataset from GC samples and patient-matched tumor-adjacent controls^32^ were utilized to evaluate the 26-gene signature in GC compared to adjacent tissues activity using the AddModuleScore function in Seurat with default parameters.

### Single-molecule fluorescent in situ hybridization

The manufacturer’s protocol was followed to perform the RNAscope HiPlex12 Reagent Kit v2 (488, 550, 650) Assay (Advanced Cell Diagnostics Cat. No. 324419). Gastric tissue sections adjacent to those used for the Visium spatial transcriptomics assay were evaluated to identify twelve RNA target genes. The tissue sections mounted on slides were baked for 1 h at 60 °C and deparaffinized in xylene and ethanol. Target retrieval was performed using a steamer at 99 °C followed by a protease treatment (Protease III for 30 min at 40 °C). Probes for the twelve genes were hybridized for 2 h at 40 °C and negative and positive control probes were run in parallel to assess sample RNA quality. A series of amplifiers were hybridized for signal amplification of single RNA transcripts for three target genes at a time which was visualized by hybridization with the first set of three cleavable fluorophores (T1-T3). Fluorophores corresponded to AF488, Dylight 550, and Dylight650. The sections were incubated with FFPE reagent for 30 min at room temperature to reduce autofluorescence and then counterstained with DAPI for 30 s. The sections were mounted, a cover slip was placed with ProLong Gold Antifade Mountant (Invitrogen, Cat. #P36930) and the section imaged using a Leica DMI 6000. After imaging, the coverslips were removed in a 4X SSC buffer (Sigma Aldrich, Cat. #SRE0068), and the fluorophores were cleaved using the cleaving solution from the kit. The sections were hybridized with the next set of fluorophores (T4-T6), incubated with FFPE reagent, counterstained with DAPI, and reimaged. This process was repeated four times until all 12 target genes were imaged. Next, hematoxylin and eosin staining was conducted. Whole slide images were acquired using Aperio AT2 whole slide scanner (Leica Biosystems Inc., IL, USA).

Imaging was performed on a Leica DMI 6000 microscope with a 40X (NA 0.85) air objective. The microscope was controlled using the Lecia Application Suite X (LAS X) v3.6.0 software. The exposure and gain were qualitatively calibrated for each channel (DAPI, Alexa Fluor-488, Dylight 550, Dylight 650) and re-adjusted each round. To capture whole-slide images of the samples, we used the Tilescan function in LAS X to automatically acquire many image tiles using the motorized stage. The image tiles were acquired with 20% overlap to enable downstream stitching into whole-slide images. These images were exported from LAS X in .lif file format.

We inputted the .lif files from each sample into MCMICRO^59^, a microscopy image processing pipeline for multiplexed images. Within the MCMICRO pipeline, we first corrected for uneven illumination by applying the BaSiC algorithm^60^. Next, we use ASHLAR^61^ to simultaneously stitch individual image tiles into whole-slide-images and register channels across multiple imaging rounds using the DAPI channel as a reference. Finally, for each sample we exported a multiplexed whole-slide OME-TIFF image containing all channels across all imaging rounds. We viewed these images with QuPath^62^ v0.5.1 and manually adjusted the channel minimum and maximum for each imaging channel across every round to minimize background noise and enhance contrast. We exported snapshots in svg format to display in figures.

## Supplementary information

Supplemental

Supplementary Data (1 - 15).

## Data Availability

Data from this study has been deposited in phs003648.v1.p1. Downloaded datasets are available under accession numbers phs001818, GSE134520, GSE150290, and PRJNA678538. Processed single-cell, spatial, and bulk RNAseq counts data, along with high-resolution H&E slides with pathologist annotations, are additionally available through the Stanford Gastric Cancer Registry Data Portal at https://gcregistry-explorer.stanford.edu.

## Code availability

The code used in this study can be accessed at https://github.com/sgtc-stanford/GIM-spatial.
