# Supplementary material for: A spatial transcriptomic signature of 26 genes resolved at single-cell resolution characterizes high-risk gastric cancer precursors
Source: NPJ Precis Oncol. 2025 Feb 25;9:52. doi: 10.1038/s41698-025-00816-w (PMC11861308; doi:10.1038/s41698-025-00816-w)
Supplement: Supplementary file 1 — Supplemental [file 41698_2025_816_MOESM1_ESM.pdf]

A spatial transcriptomic signature of 26 genes resolved at single-cell resolution characterizes high-risk gastric cancer precursors

AUTHORS

Robert J. Huang[1*], Ignacio A. Wichmann[2,3,4*], Andrew Su[5], Anuja Sathe[2], Miranda V. Shum[1], Susan M. Grimes[2], Rithika Meka[2], Alison Almeda[2], Xiangqi Bai[2], Jeanne Shen[6], Quan Nguyen[5], Ingrid Luo,[7] Summer S. Han[7,8,9], Manuel R. Amieva[10,11], Joo Ha Hwang[1], Hanlee P. Ji[2]

***These authors contributed equally to this manuscript.**

[1]Division of Gastroenterology, Department of Medicine, Stanford School of Medicine, Stanford, CA, 94305, USA

[2]Division of Oncology, Department of Medicine, Stanford School of Medicine, Stanford, CA, 94305, USA

[3]Division of Obstetrics and Gynecology, Department of Obstetrics, Escuela de Medicina, Pontificia Universidad Católica de Chile, Santiago, 8331150, Chile

[4]Advanced Center for Chronic Diseases (ACCDiS), Pontificia Universidad Católica de Chile, Santiago, 8331150, Chile

[5]Institute for Molecular Bioscience, The University of Queensland, Brisbane, QLD, 4072, Australia

[6]Department of Pathology, Stanford School of Medicine, Stanford, CA, 94305, USA

[7]Quantitative Sciences Unit, Department of Medicine, Stanford School of Medicine, Stanford, CA, 94305, USA

[8]Department of Neurosurgery, Stanford School of Medicine, Stanford, CA, 94305, USA

[9]Stanford Cancer Institute, Stanford, CA, 94305, USA

[10]Department of Microbiology and Immunology, Stanford University, Stanford, CA, 94305, USA

[11]Department of Pediatrics, Stanford University, Stanford, CA, 94305, USA

To whom correspondence should be addressed.

Hanlee P. Ji

Email: genomics_ji@stanford.edu

Joo Ha Hwang

Email: jooha@stanford.edu

Supplementary Figure 1

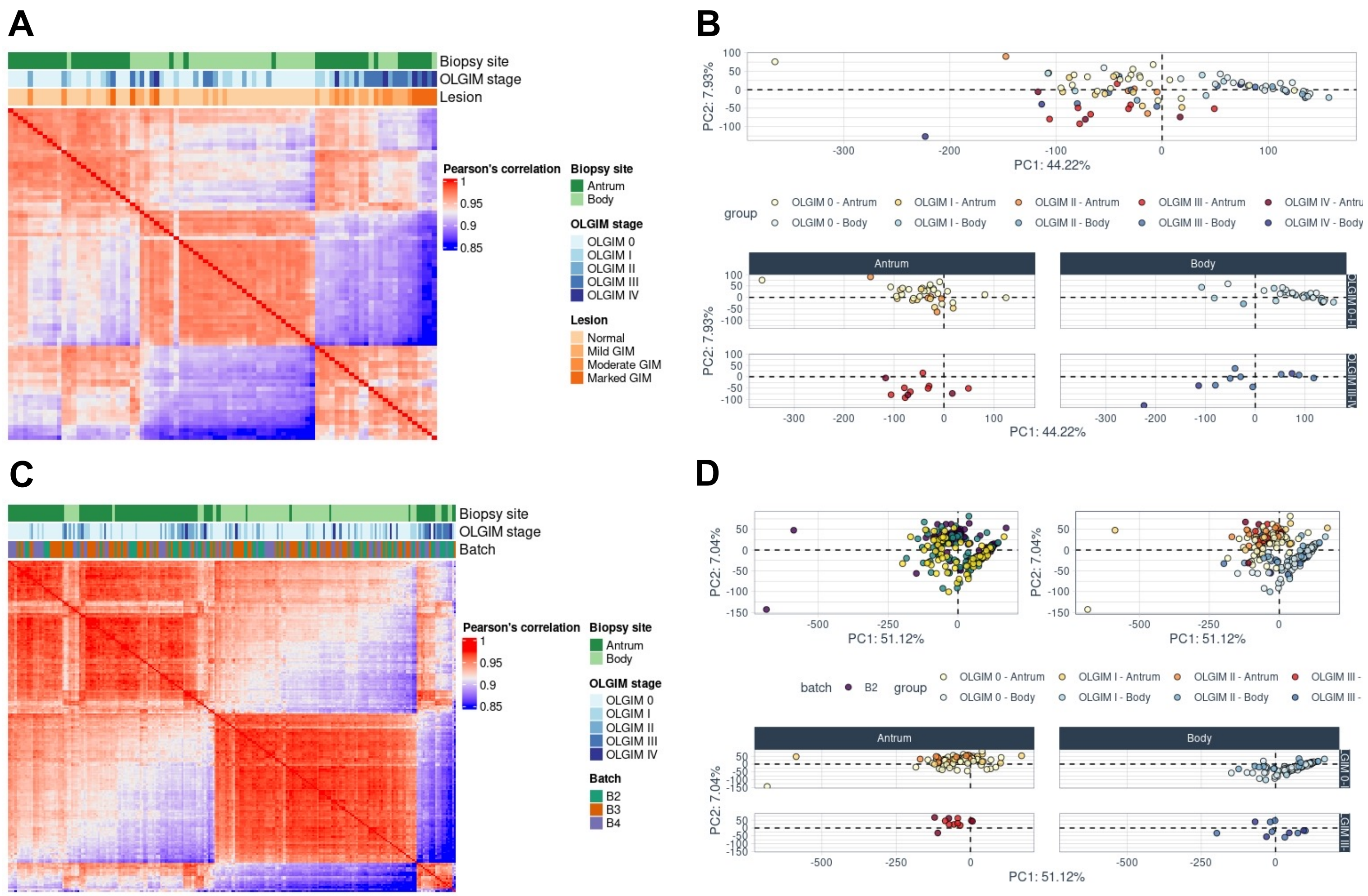

Supplementary Figure 1. Unsupervised clustering. A) Hierarchical clustering using pairwise Pearson's correlation coefficient between samples for the discovery cohort (N=88 samples: 22 high-risk, 66 low-risk). Complete linkage clustering method (default) was used. Preferential grouping of OLGIM III and IV samples is observed, regardless of anatomic site. OLGIM 0, I and II cases group preferentially by anatomic site of the biopsy (*e.g.*, body or antrum). B) Principal components analysis showing preferential grouping OLGIM III and IV samples regardless of anatomic sites. OLGIM 0, I and II samples tend to cluster by anatomic site consistent with hierarchical clustering approach. C) and D) Hierarchical clustering and principal components analysis between samples for the held-out validation cohort (N=215 samples: 22 high-risk, 193 low-risk). Sequencing batch was added. No evident batch effects were observed by either method. B1: Batch 1, B2: Batch 2, B3: Batch 3.

Supplementary Figure 2

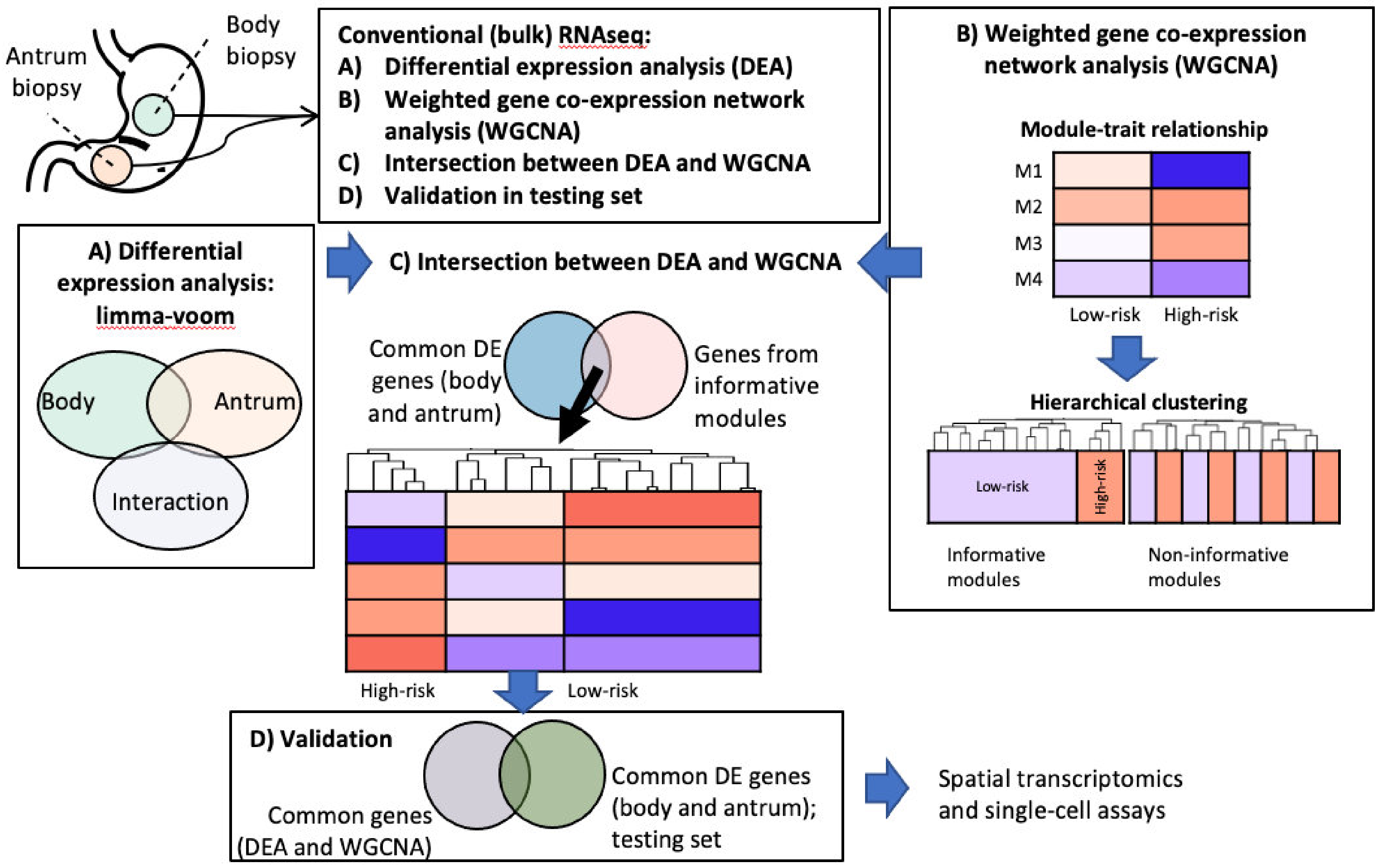

Supplementary Figure 2. Analysis strategy for bulk RNA-seq data. A) Differential expression analysis (discovery cohort). Samples grouped as high-risk (OLGIM III and IV) were compared with low-risk (OLGIM 0, I and II) in the antrum and body, separately. An interaction term was calculated by for genes which differential expression profile differed significantly by anatomic location (interaction term). Common differentially expressed genes in the body and antrum, excluding genes with a significant interaction term, were kept for downstream analysis. B) Weighted gene co-expression network analysis (WGCNA). WGCNA was conducted using top 15% most variable genes. Gene co-expression modules were identified. The first principal component of each gene module was calculated to compare high- and low-risk groups (module-trait relationship). Hierarchical clustering using scaled expression levels, Pearson correlation distance and Ward's clustering method was performed to inspect gene modules with module-trait relationship indicative of differences between high- and low-risk samples. Gene modules that clustered high-risk samples apart from low-risk samples were considered informative and kept for downstream analyses. C) Intersection between DEA and WGCNA. A total of 399 differentially expressed genes from A) were intersected with 815 genes from B). A curated set of 314 co-expressed and differentially expressed genes was identified. Based on gene clusters and Z-score, we selected a subset of 105 genes for further evaluation in the validation cohort. D) Differential expression was conducted as in A) in a held-out validation cohort of 215 samples. Differentially expressed genes from this validation cohort were intersected with the 105 genes from C). This resulted in the validation of a highly curated set of 100 genes (95.23% of the original genes).

Supplementary Figure 3

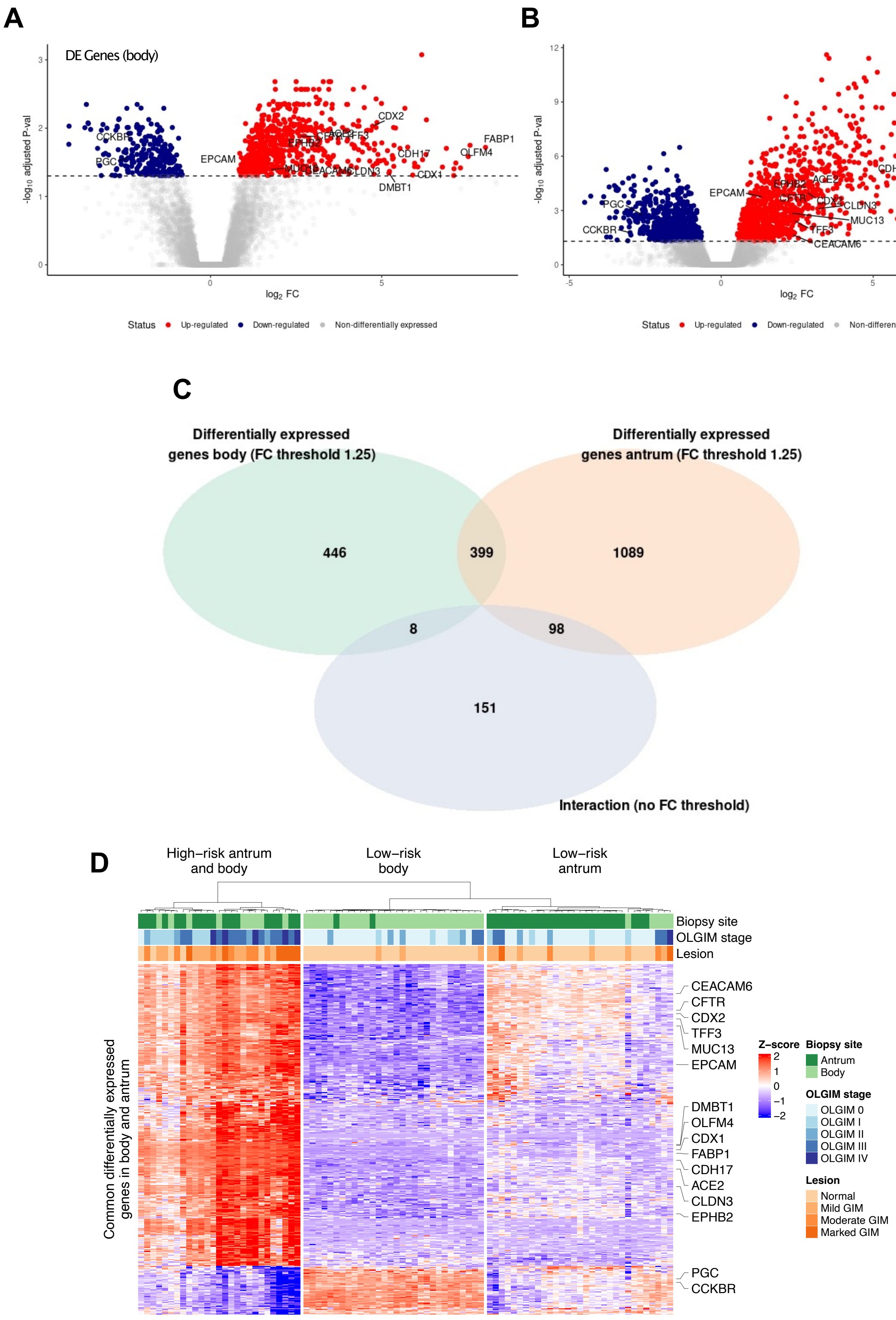

Supplementary Figure 3. Differential expression analysis, discovery cohort. A-B) Volcano plot showing significantly upregulated (red) and downregulated (blue) genes in the body (A) and antrum (B). *C*) Differentially expressed genes (DEGs) between high- and low-risk OLGIMs were defined using a fold-change threshold of 1.25 and adjusted p-value ≤ 0.05. DEGs for the body and antrum were identified. We further confirmed that this these intersected genes did not demonstrate any significant statistical interaction with anatomic location within the stomach. This analysis resulted in 399 genes. D) Heatmap and hierarchical clustering using Pearson distance and Ward clustering method of 399 differentially expressed genes common to the body and antrum. *A-D:* Known intestinal metaplasia and gastric epithelial markers are labelled.

Supplementary Figure 4

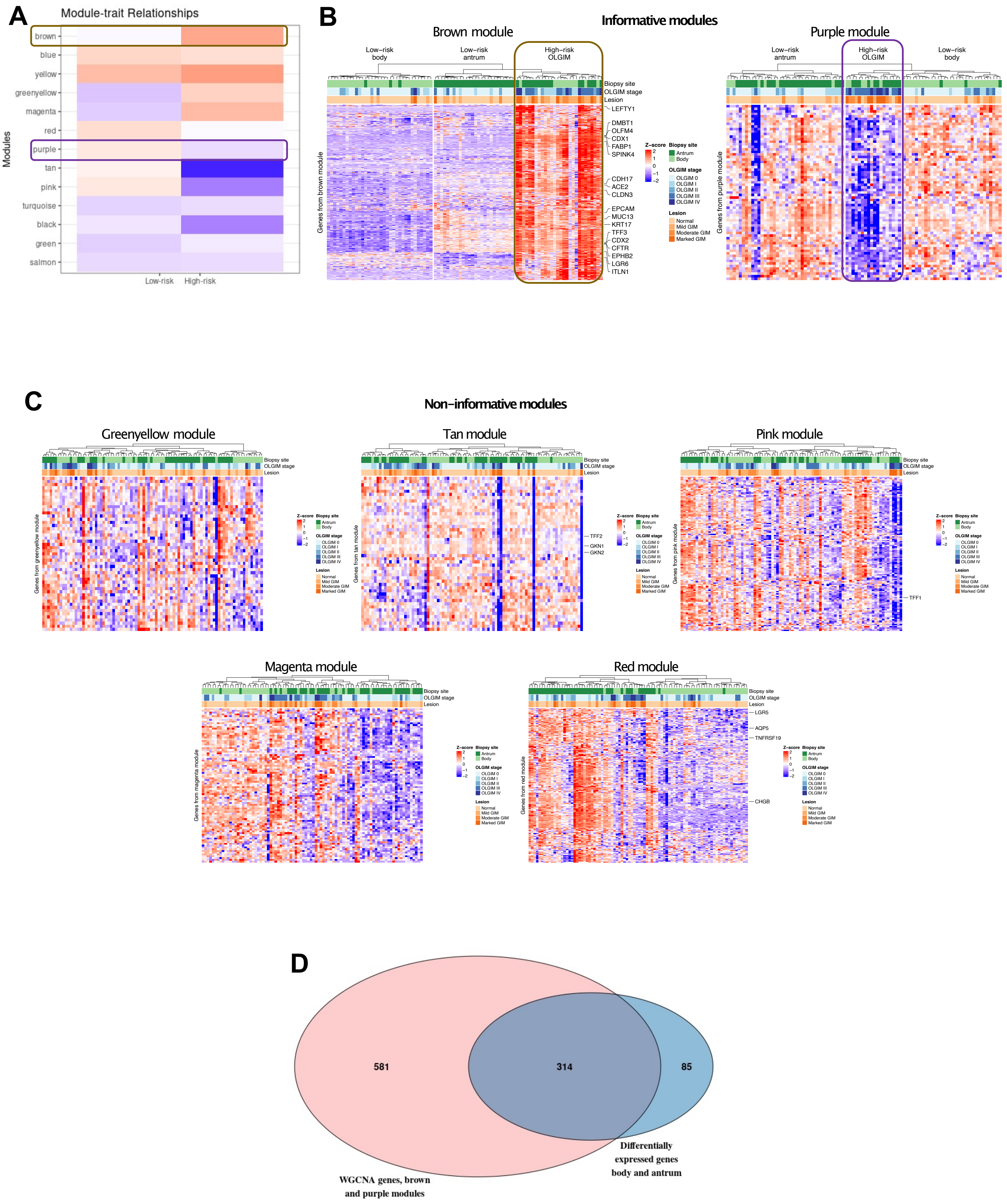

Supplementary Figure 4. Weighted gene co-expression network analysis (WGCNA). A) Module-trait relationship showing Z-scaled Eigen gene value (first principal component of each gene module) averaged across samples from high- and low-risk groups. Brown, green-yellow and magenta modules are increased in high-risk GIM. Red, purple, tan and pink modules are reduced in high-risk GIM. B-C) Hierarchical clustering of samples from brown and purple modules using Pearson distance and Ward clustering method. These modules show preferential clustering of high-risk GIM. The brown module captures most metaplasia and gastric epithelial cell markers, that are over-expressed in high-risk GIM. D-H) Non-informative gene modules from WGCNA. These modules do not segregate high-risk GIM. I) Intersection of genes from the brown and purple modules with results from differential expression analysis.

Supplementary Figure 5

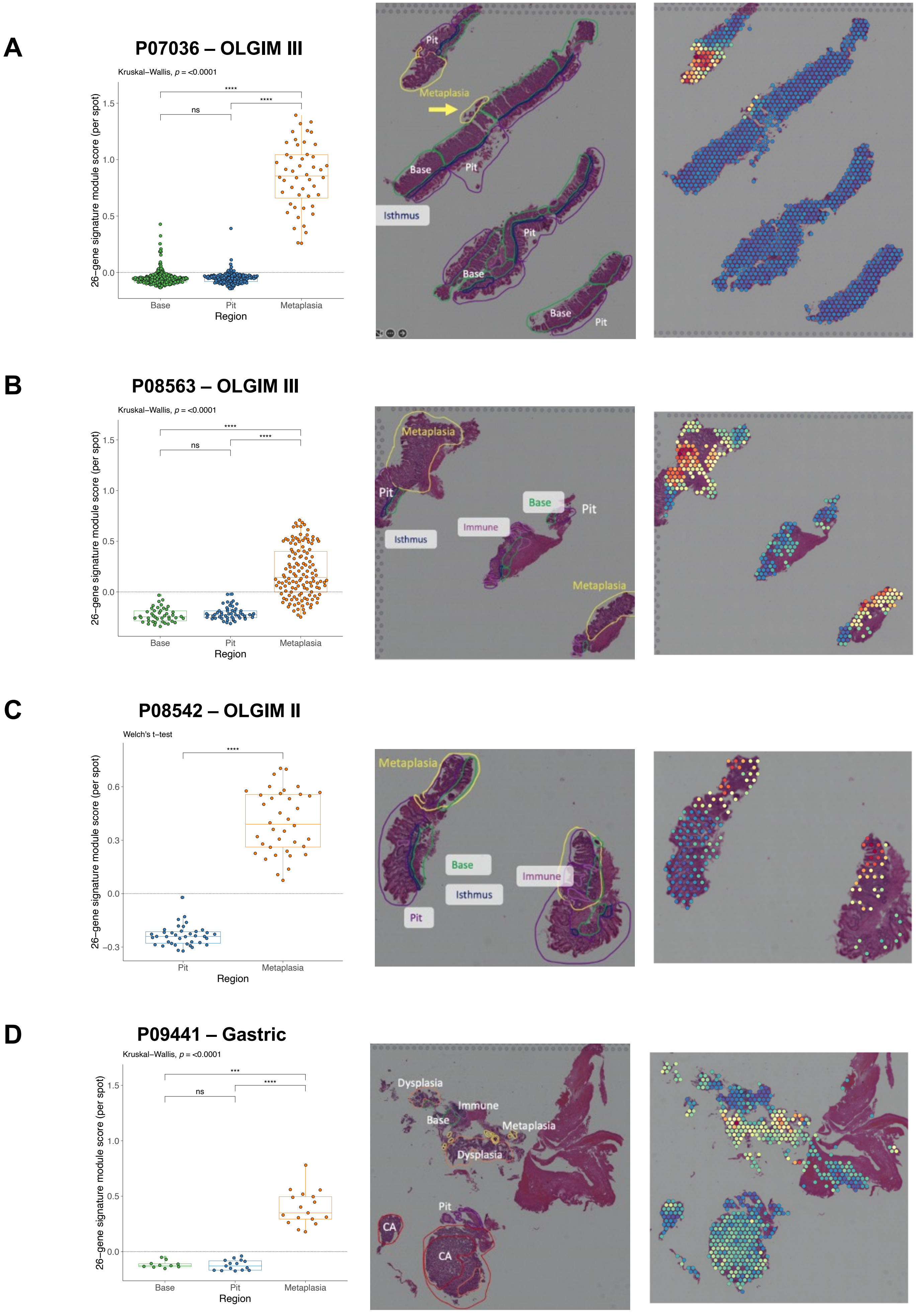

Supplementary Figure 5. Spatial mapping of the module score. The 26-module score value was calculated for each individual spot from 5 samples undergoing spatial transcriptomics assay. Statistically significant differences were observed between metaplastic foci and normal gland base and pit across all samples (Kruskal-Wallis followed by Dunn's test). Spatial mapping of the signature was highly consistent with pathologist annotations of intestinal metaplasia.

Supplementary Figure 6

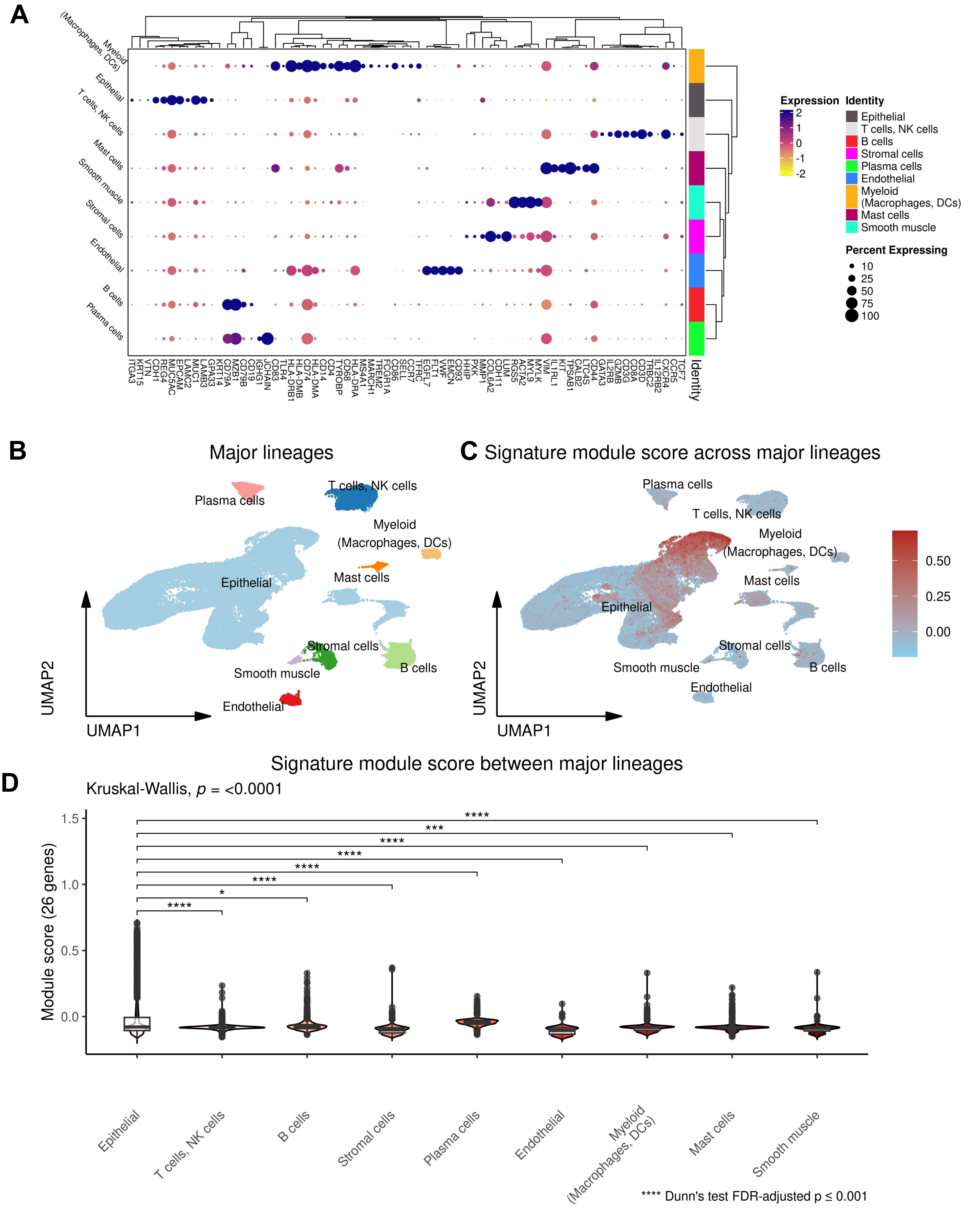

Supplementary Figure 6. Signature module score is increased in epithelial cells. A) Clustered dotplot showing markers for major lineages. B) UMAP plot showing 9 major cell lineages. C) Signature module score plotted across UMAP plot. D) Module score comparison between epithelial cells and other major cell lineages. Kruskal-Wallis followed by Dunn’s test.

Supplementary Figure 7

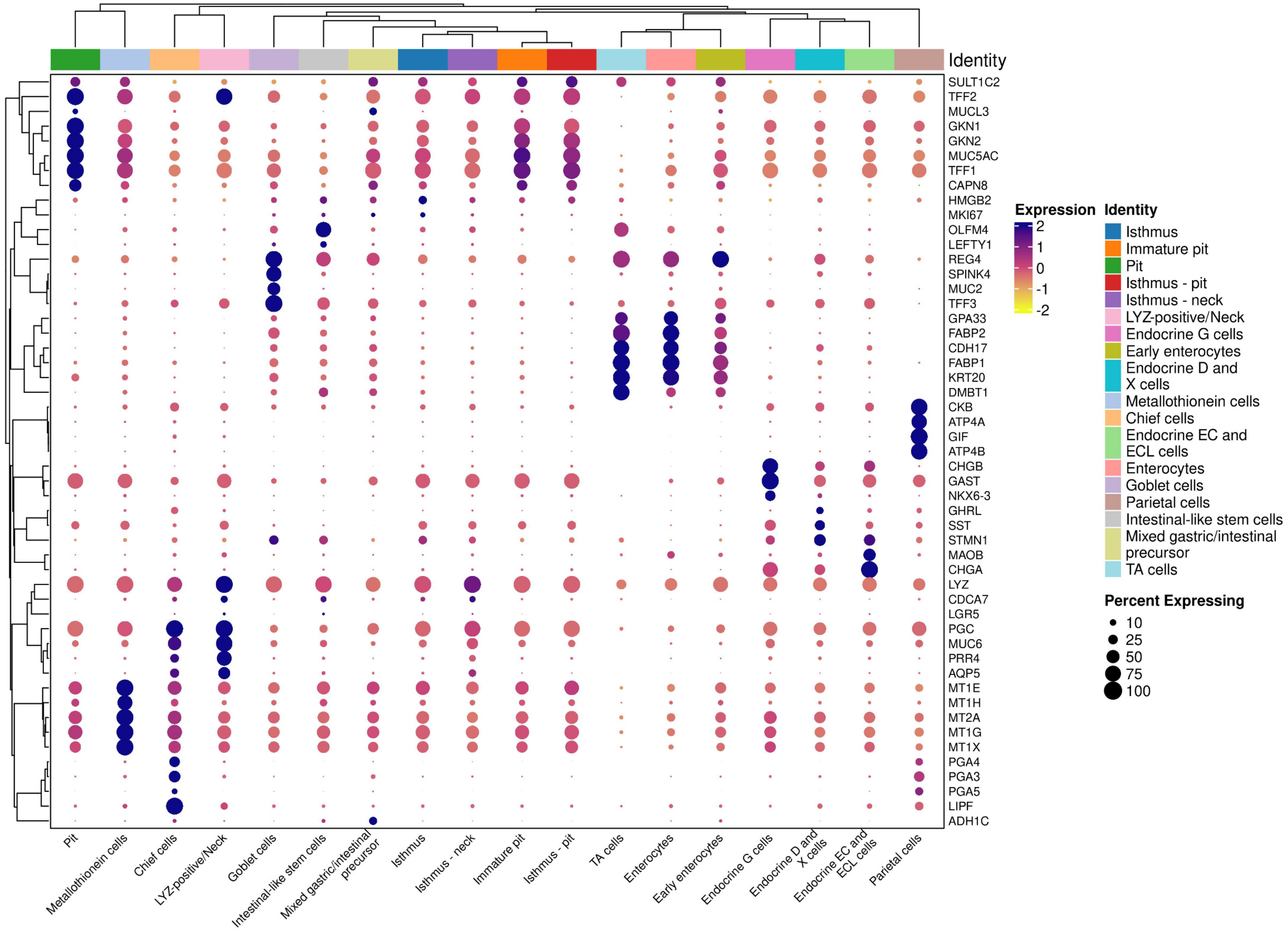

Supplementary Figure 7. Epithelial cell cluster markers. Clustered dot plot showing markers for minor lineages from the gastric epithelium.

Supplementary Figure 8

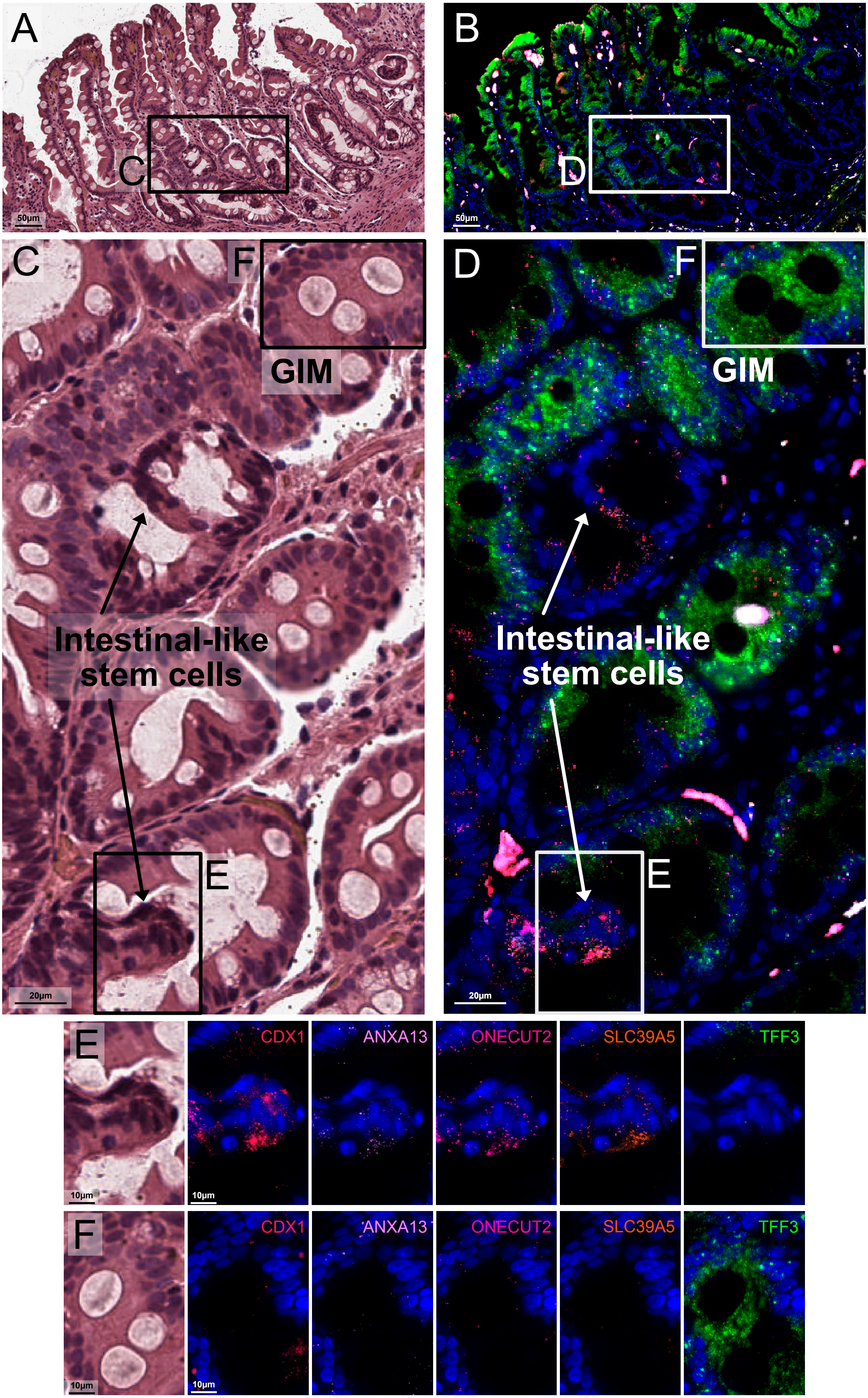

Supplementary Figure 8. Single-molecule fluorescence in situ hybridization (smFISH) of gastric intestinal metaplasia. A) and B): Representative region showing H&E staining and smFISH, respectively, for five genes in a gastric intestinal metaplasia foci from sample P08563 (OLGIM stage III): *CDX1*, *ANXA13, ONECUT2, SLC39A5,* and *TFF3.* C) and D): Inset magnification for highlighted area from A) and B), respectively (90-degree clockwise rotation). E): Highlighted regions in C) and D), showing H&E and individual channels from areas enriched for poorly-differentiated columnar cells near the isthmic/crypt regions in the metaplastic glands (intestinal-like stem cells). These cells show elevated expression of *CDX1*, *ANXA13, ONECUT2* and *SLC39A5*. F): Highlighted regions in C) and D), showing H&E and individual channels from areas enriched for well-differentiated (mature) cells from the GIM foci. These cells show elevated expression of *TFF3*.

Supplementary Figure 9

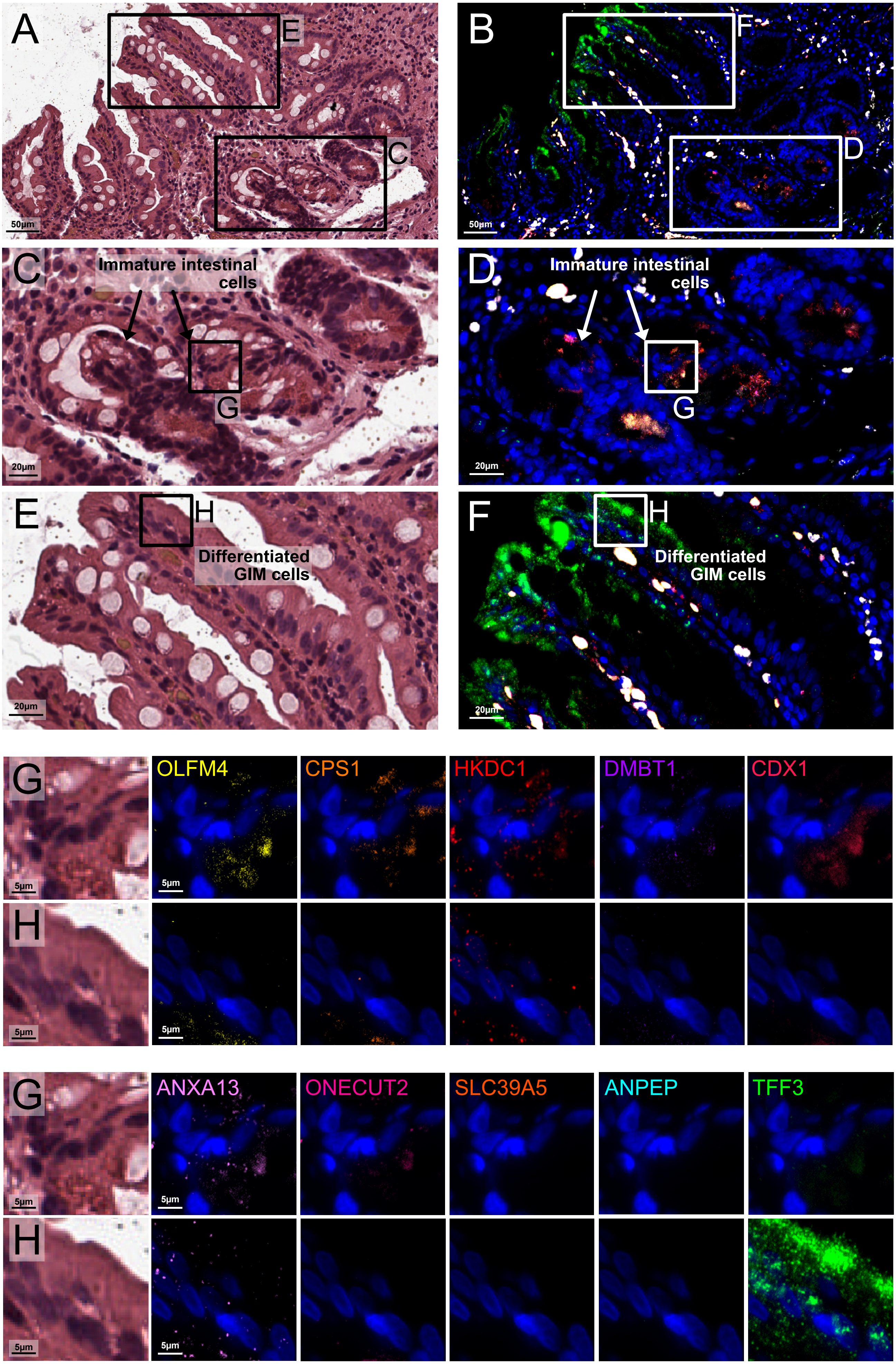

Supplementary Figure 9. Single-molecule fluorescence in situ hybridization (smFISH) of gastric intestinal metaplasia. A) and B): Representative region showing H&E staining and smFISH, respectively, for ten genes in a gastric intestinal metaplasia foci from sample P08542 (OLGIM stage II, antrum): *OLFM4, CPS1, HKDC1, DMBT1, CDX1, ANXA13, ONECUT2, SLC39A5, ANPEP,* and *TFF3*. Panels C) and E) represent magnified H&E images, and D) and F) represent corresponding smFISH, respectively. G) depicts an area enriched in immature intestinal cells, with elevated expression of *OLFM4*, *CPS1, DMBT1, CDX1* and *ONECUT2*. H) depicts an area of mature, well-differentiated cells characterized by strong expression of *TFF3*. *HKDC1* is expressed in both areas, with relatively higher expression among the immature cells.

Supplementary Figure 10

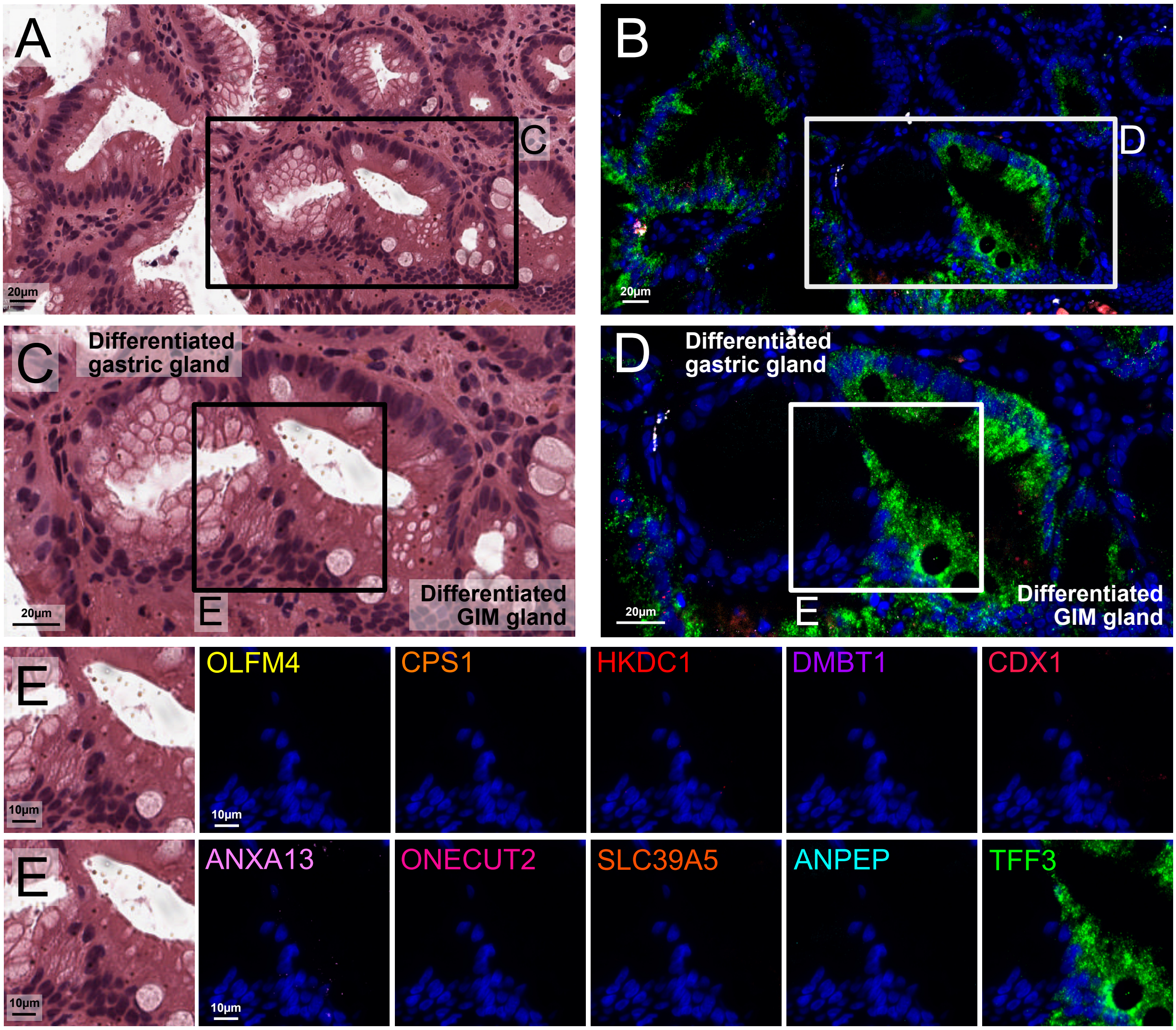

Supplementary Figure 10. Single-molecule fluorescence in situ hybridization (smFISH) of well-differentiated gastric intestinal metaplasia. A) and B): H&E and corresponding RNAscope smFISH data from a transverse section of metaplasia from patient P07036 (OLGIM II, antrum). C) and D): Inset magnification for highlighted area from A) and B), respectively. E) represents an area of well-differentiated (mature) GIM. Ten genes from the signature are shown in the merged smFISH panels: *OLFM4, CPS1, HKDC1, DMBT1, CDX1, ANXA13, ONECUT2, SLC39A5, ANPEP* and *TFF3*.

Supplementary Figure 11

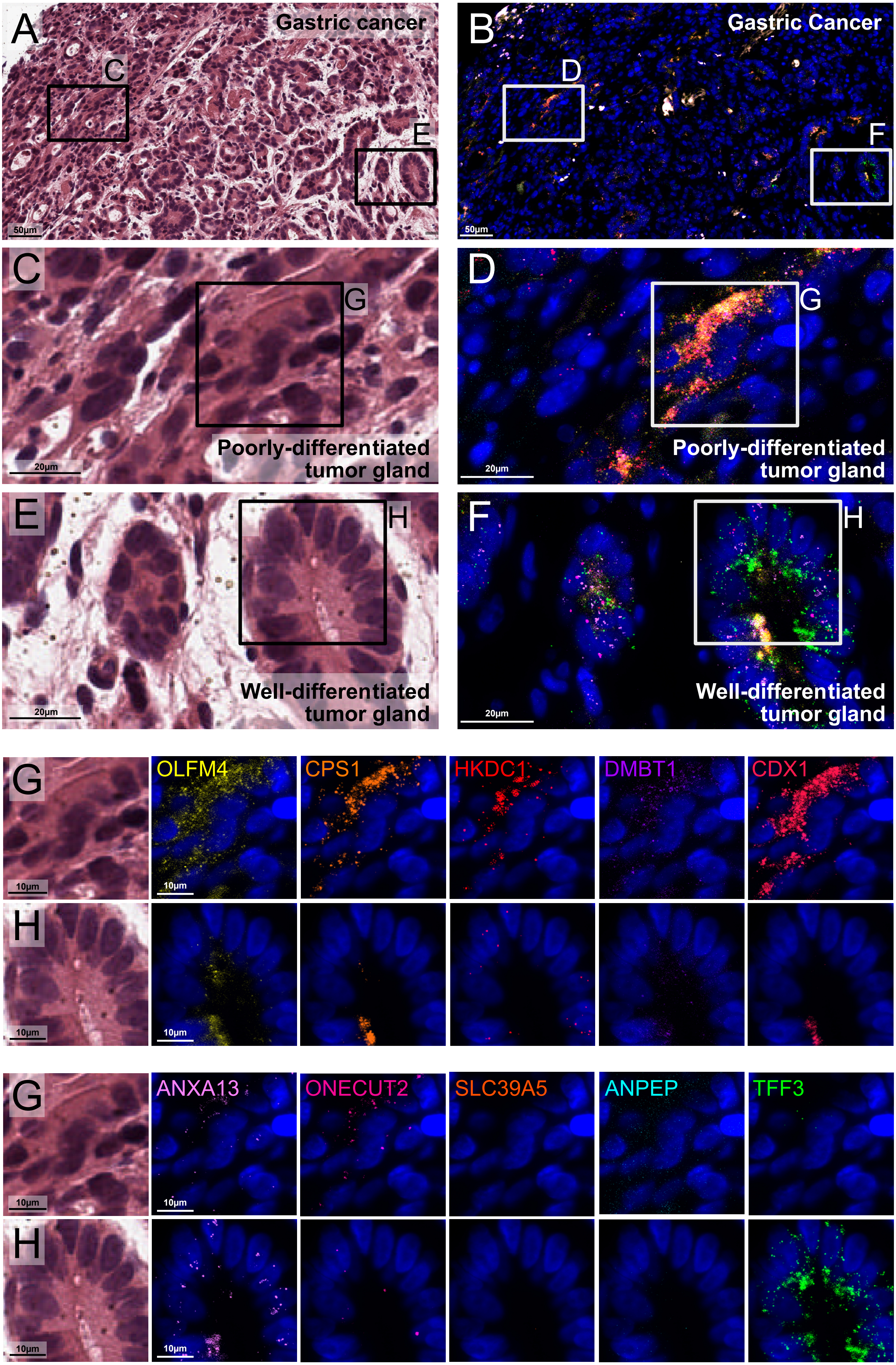

Supplementary Figure 11. Single-molecule fluorescence in situ hybridization (smFISH) of gastric cancer. H&E and corresponding RNAscope smFISH from patient P09441 (intestinal-type early gastric cancer, *incisura angularis*). Ten genes from the signature are shown in the merged smFISH panels: *OLFM4, CPS1, HKDC1, DMBT1, CDX1, ANXA13, ONECUT2, SLC39A5, ANPEP* and *TFF3*. A) (H&E) and B) (smFISH) depict a wide-field view of a portion of the tumor with well-differentiated tumor glands to the right and poorly-differentiated tumor glands to the left, at the same magnification. C) and D) depict the poorly-differentiated sections of the tumor, whereas E) and F) depict the well-differentiated glands of the tumor. G) On magnification, the poorly-differentiated glands display increased expression of *OLFM4, CPS1, HKDC1, DMBT1* and *CDX1*. H) By contrast, the well-differentiated glands express *TFF3*. *ANXA13* and *ONECUT2* are expressed similarly between both regions.

SUPPLEMENTARY DATA LEGEND

Supplementary Data 1: Overview of specimen sourcing from cohorts. GAPS, GAstric Precancerous conditions Study; TCGA, The Cancer Genome Atlas; smFISH, single molecule fluorescence in situ hybridization; GIM, gastric intestinal metaplasia; GC, gastric cancer.

Supplementary Data 2: Samples from GAPS. OLGIM, operative link on gastric intestinal metaplasia.

Supplementary Data 3: Samples from TCGA. TP, primary tumor; NT, adjacent control.

Supplementary Data 4: ScRNA-seq samples. NAG, non-atrophic gastritis; CAG, chronic atrophic gastritis; EGC, early gastric cancer.

Supplementary Data 5: Spatial cohort samples.

Supplementary Data 6: Genes from bulk RNA discovery.

Supplementary Data 7: Genes from bulk RNA validation.

Supplementary Data 8: Genes from spatial transcriptomics pseudo-bulk analysis.

Supplementary Data 9: Genes from TCGA validation.

Supplementary Data 10: Enrichment for gene ontology terms.

Supplementary Data 11: Enrichment for cell type signatures.

Supplementary Data 12: ScRNA-seq Comparison between cell types.

Supplementary Data 13: ScRNA-seq Comparison between epithelial cell types.

Supplementary Data 14: ScRNA-seq Comparison between stages of Correa’s cascade.

Supplementary Data 15: ScRNA-seq Comparison between tumor and adjacent tissue.
